# Wound healing and Cadmium detoxification in the earthworm *Lumbricus terrestris* – a potential case for coelomocytes?

**DOI:** 10.3389/fimmu.2023.1272191

**Published:** 2023-12-05

**Authors:** Gerhard P. Aigner, Veronika Peer, Birgit Fiechtner, Cláudio Adriano Piechnik, Martina Höckner

**Affiliations:** Department of Zoology, Center for Molecular Biosciences, University of Innsbruck, Innsbruck, Austria

**Keywords:** Annelida, stress, injury, amputation, innate immunity, immune cells, metallothionein, toll-like receptors

## Abstract

Earthworms are affected by physical stress, like injury, and by exposure to xenobiotics, such as the toxic metal cadmium (Cd), which enters the environment mainly through industry and agriculture. The stress response to the single and the combination of both stressors was examined in regenerative and unharmed tissue of *Lumbricus terrestris* to reveal if the stress response to a natural insult like injury (amputation) interferes with Cd detoxification mechanisms. We characterized the roles of metallothionein 1 (MT1) and MT2 isoforms, heat shock protein 70 as well as immune biomarkers such as the toll-like receptors (TLR) single cysteine cluster TLR and multiple cysteine cluster TLR. The role of the activated transcription factors (ATFs) ATF2, ATF7, and the cAMP responsive element binding protein as putative regulatory intersection as well as a stress-dependent change of the essential trace elements zinc and calcium was analyzed. Phosphorylated AMP activated protein kinase, the cellular energy sensor, was measured to explore the energy demand, while the energy status was determined by detecting carbohydrate and protein levels. Taken together, we were able to show that injury rather than Cd is the driving force that separates the four treatment groups – Control, Cd exposure, Injury, Cd exposure and injury. Interestingly, we found that gene expression differed regarding the tissue section that was analyzed and we hypothesize that this is due to the migration of coelomocytes, earthworm immune cells, that take over a key role in protecting the organism from a variety of environmental challenges. Surprisingly, we discovered a role for MT1 in the response to multiple stressors and an isoform-specific function for the two newly characterized TLRs. In conclusion, we gathered novel information on the relation of innate immunity, wound healing, and Cd detoxification mechanisms in earthworms.

## 1 Introduction

Earthworms inhabit most of the Earth’s terrestrial ecosystems (1), and their soil-dwelling lifestyle makes them vulnerable to both environmental and anthropogenic sources of stress. They have therefore developed a strong stress response and a potent innate immune system. The latter is composed of a humoral and a cellular part, so-called coelomocytes, which consist of different cell types located in the coelomic cavity. Upon external stimulation coelomocytes are extruded through dorsal pores consequently covering and protecting the earthworm body from external challenges that can range from soil pathogens to xenobiotics (2, 3).

One of the major xenobiotics, which is brought into the environment is the toxic metal cadmium (Cd) with an estimated amount of >5.5 g Cd ha^−1^ yr^−1^ in Europe (4). Cd concentrations in European soils vary depending on the history of land use. In France, for example, Cd concentrations range from a median of 0.13 mg/kg dry soil for forest areas to a median of 1.3 mg/kg for soils derived from urban, mining, industrial or military areas, where locally extreme values of up to 162 mg/kg dry soil were detected (5). Cd is a proven carcinogen and is able to substitute zinc (Zn) and calcium (Ca) due to the same charge and ionic radius (ionic mimicry), which allows Cd to hijack transport mechanisms across the membrane (6). Subsequently, Cd exhibits both immune- and cytotoxic effects, interfering with a broad range of processes from epigenetic mechanisms to energy metabolism (7–9). However, organisms have developed protection strategies to counteract adverse effects of cellular stress, such as modulating the expression of heat shock proteins (HSPs) (10, 11) and more specifically in response to Cd, the production of so-called metallothioneins (MTs) (12). Three isoforms (MT1-3) have been identified in *Lumbricus rubellus*. The most studied isoform is the Cd-inducible MT2, which is responsible for Cd detoxification. On the contrary, MT1 has been suggested to function in metal homeostasis (13) and a third isoform, MT3, has been found mainly in embryonic tissues (14). Recently, also non-MT proteins have been discovered that function in Cd detoxification (15).

Coelomocytes take over key roles after physical stress (like injury), in regeneration, and wound healing. The regenerative capacity differs largely in the annelid phylum (16). It seems that also gene expression during regeneration is species-specific as shown in *Lampito mauritii*, *Drawida calebispecies* (17), and *Eisenia fetida* (18). Furthermore, earthworm transcriptome studies reveal transcripts that cannot be assigned to any homologs from other species, although there have been overlaps with genes expressed in vertebrate model organisms like zebrafish and mouse (17, 18). Therefore, we see a high potential in studying earthworms in more detail to unearth novel regenerative mechanisms. Regeneration can be defined as periodic replacement of cells and tissue, the regeneration of lost body parts or tissue is called post-traumatic regeneration (19). According to Kostyuchenko and Kozin, the latter includes processes that can partially overlap, like closure and wound healing; immune and/or nonspecific defense response; recruitment of cells that are to form a regenerate; growth of the regenerate; patterning and differentiation. Annelid regeneration generally involves cell proliferation and the formation of a blastema, which is a mass of undifferentiated cells. There are two ways to close a wound described in *E. fetida*, namely alimentary tract eversion and formation of a coelomic plug (20). A study on wounded *Dendrobaena veneta* revealed that irradiation led to wound closure by muscle contraction (21).

It has already been shown that coelomocytes accumulate in the regenerating blastema in *Eisenia andrei*. The latter study also revealed that specific subsets of coelomocytes form clusters in the blastema, while others were not only present in the blastema but also in the body wall, alimentary canal and in the muscle, probably to remove damaged cells (22). A previously published study suggested that wound healing and the detoxification of Cd are cellular processes that interact with each other, since in amputated earthworms the expression of MT2 was drastically decreased (23). Few information is available on *Lumbricus terrestris* (24) wound healing or regeneration specifically related to Cd exposure. By introducing a three-sided cut into the integument posterior to the clitellum, it has been suggested that Cd impairs enzymes that are responsible for wound healing in *L. terrestris* (25). There are several studies on the effects of polychlorinated biphenyls (PCBs), which showed delayed healing effects and accelerated allograft rejection (26, 27).

Pattern recognition receptors (PRRs) activate the immune system as a first response to and protection against pathogens (28). The best known PRRs are the Toll-like receptors (TLRs), which consist of three domains – a leucine-rich repeat extracellular domain, a transmembrane domain, and an intracellular Toll/IL-1 receptor (TIR) domain (29). TLR-mediated downstream signaling cascades are known to activate transcription factors of the cAMP responsive element binding protein (CREB)/activating transcription factor (ATF) family, subsequently leading to the induction of genes related to innate immunity and cell survival (30, 31). In earthworms, the exact number of CREB/ATF proteins and TLRs is unknown. Two TLRs have been characterized in *E. andrei*, a single cysteine cluster (scc) TLR and a multiple cysteine cluster (mcc) TLR, which are mainly expressed in reproductive organs and could be linked to gregarine infection (32, 33). Gram-positive bacteria induced the expression of sccTLR in *E. andrei* coelomocytes (32).

The effect of Cd on energy allocation has been evaluated in *Enchytraeus albidus*, showing that after four to eight days, carbohydrate reserves were significantly reduced, however, protein levels showed no change (34). AMP activated protein kinase (AMPK), the metabolic checkpoint in the cell, could provide valuable clues about the metabolic state of a tissue. In the activated form (phosphorylated) it is involved in regulating cell growth, apoptosis, glucose and lipid metabolism (35). Upon energy stress AMPK inhibits anabolic or stimulates catabolic pathways and it is also known to increase the number of mitochondria (36, 37). Little information is available on the energy demands in earthworms during wound healing or regenerative processes.

The Cd stress response, PRRs, regulatory factors, essential trace elements as well as the energy metabolism were evaluated to reveal factors that might be related to both stressors, revealing a putative link between wound healing and detoxification mechanisms. Furthermore, as mentioned above, we analyzed the suppression of Cd-induced MT2 expression in amputated earthworms as previously suggested (23).

## 2 Materials and methods

### 2.1 Experimental setup and sampling

The earthworm species *L. terrestris* and soil (mixture of humus and peat) were ordered from Proinsects GmbH (Minden, Germany). For the experimental setup, amputated and unharmed earthworms were kept in Cd-contaminated and non-contaminated soil, leading to a total of four treatment groups. Earthworms were amputated by excising ~1 cm of their posterior end with a sharp scalpel directly before the start of the experiment. The group Cd_Cut consisted of amputated earthworms that were kept in soil spiked with 50 mg CdCl_2_/kg dry soil, whereas unharmed individuals in Cd-contaminated soil were defined as the Cd group. Amputated individuals in non-contaminated soil were considered the Cut group and unharmed earthworms in non-contaminated soil served as control. All groups were fed once a week with 0.9 g of horse manure and were kept at 75% soil humidity, 15°C room temperature and a day/night cycle of 12/12 h. Before the start of the experiment, all earthworms were stored in control soil for at least two weeks for acclimation.

Tissue samples were taken once a week over a total of three weeks. In amputated earthworms the regenerative tissue (Tissue 1) and the anterior section (Tissue 2) were sampled. In unharmed earthworms the tissue sections corresponding to the Cut groups were sampled. The number of biological replicates is shown in **Supplementary Table S1**. Tissue samples for RNA extraction were stored in 1 ml absolute Ethanol (Merck, Darmstadt, Germany) at -20°C. For Zn quantification and protein extraction, tissue samples were shock frozen in liquid nitrogen and stored at -80°C until further use. For Ca quantification, tissue was processed immediately after sampling.

### 2.2 RNA extraction and cDNA synthesis

For RNA extraction, each tissue sample was placed in 1.5 ml screw-cap tubes containing 1 ml TRI REAGENT^®^ (Sigma Aldrich, St. Louis, MO, USA) and RNase-free glass beads. Tissue was disrupted using a FastPrep-24™ 5G benchtop homogenizer (MP Biomedicals, Santa Ana, CA, USA). The following settings were applied for homogenization: three cycles of 40 s at 6 m/s with a pause of 120 s between each cycle. RNA extraction and DNA digestion were performed as described in the user manual. DNA was digested using DNAse I (Thermo Fisher Scientific, Waltham, MA, USA), including the RNAse inhibitor RiboLock (Thermo Fisher Scientific, Waltham, MA, USA). The quality of the extracted RNA was checked by gel electrophoresis to assess the integrity of the rRNA bands. RNA concentration was measured in technical triplicates using the Quant-iT™ RiboGreen™ RNA Assay Kit (Thermo Fisher Scientific, Waltham, MA, USA) on a Victor X4 plate reader (Perkin Elmer, Waltham, MA, USA). 450 ng of total RNA was reverse transcribed using RevertAid™ H Minus Reverse Transcriptase (Thermo Fisher Scientific, Waltham, MA, USA), Random Hexamer Primers (Thermo Fisher Scientific, Waltham, MA, USA), and RiboLock (Thermo Fisher Scientific, Waltham, MA, USA) according to the manufacturer’s instructions.

### 2.3 Sequence generation

For sequence generation of *L. terrestris* sccTLR, a previously published CD842 primer pair from *D. veneta* was used (38). PCRs were performed on a T 100 thermal cycler (Bio-Rad, USA) using Titanium Taq DNA polymerase (Takara, Japan). After gel electrophoresis, bands were excised and were purified using the QIAquick Gel Extraction Kit (Qiagen GmbH, Netherlands) according to the manufacturer’s instructions. TA cloning was conducted with the purified PCR products using the pGEM^®^-T vector systems (Promega, USA) as described in the manual. Colony PCR was performed using SP6 and T7 primer sequences. PCR products were purified (QIAquick PCR Purification Kit, Qiagen, Netherlands) and were then sent for sequencing (Microsynth, Switzerland).

We used homolog sequences to search the NCBI Blast+ assembled RNA-Seq database of *L. terrestris* to find sequences for mccTLR, MT1, CREB, ATF2, and ATF7 and to design primers (**Supplementary Table S2**). Amino acid sequences were analyzed using the SMART™ online protein domain characterization tool. Sequences (**Supplementary Table S3**) were published in Genbank under the following Accession Numbers: sccTLR (OR596221), mccTLR (OR596222), MT1 (OR596220), ATF2 (OR610157), ATF7 (OR610158), CREB (OR610159).

### 2.4 Quantitative real-time PCR

A primer matrix was applied to determine optimal primer concentrations and a standard curve was generated from a series of dilutions of known template concentrations to quantify absolute RNA copy numbers. Copy numbers were measured using a QuantStudio™ 3 Real-Time PCR system (Thermo Fisher Scientific, Waltham, MA, USA). The list of primer sequences used for qPCR is given in **Supplementary Table S2**. 5 µl of Power SYBR™ Green PCR Master Mix (Thermo Fisher Scientific, Waltham, MA USA) was used per sample. CREB, ATF2, ATF7, MT2, sccTLR and mccTLR were amplified as follows: 1 µl 10 × BSA, 1 µl forward primer (9 µM), 1 µl reverse primer (9 µM) and 2 µl cDNA. MT1 was amplified as described above, but using a reverse primer concentration of 3 µM. qPCRs were conducted in technical triplicates using the following settings: 50°C for 2 min; 95°C for 10 min; 40 repeats of 95°C for 15 s and 60°C for 1 min. Absolute copy numbers per 100 ng RNA were determined from the standard curve. Values were normalized to the threshold. Values below the detection range of our standard curve were set to zero.

### 2.5 Zinc quantification

Total Zn levels were quantified using the Zinc Quantification Kit (Fluorometric) (ab176725, Abcam^®^, United Kingdom). In detail, tissue samples were weighed and placed in microcentrifuge screw-cap tubes containing 250 µl EDTA-free lysis buffer [50 mM Tris, 100 mM NaCl, 1 mM sodium vanadate, 0.5 mM PMSF, 1 µg/ml aprotinin, 1 µg/ml leupeptin, 1 µg/ml pepstatin, 10 µM MG132, 0.5 mM DTT] and glass beads. Tissue was disrupted as described for the RNA extraction method. After homogenization, a centrifugation step was performed at 4°C for 1 min at the maximum speed to eliminate air bubbles. 250 µl of 7% TCA solution was added to the sample, vortexed and centrifuged at 4°C for 5 min at maximum speed. 400 µl of the supernatant were transferred to a new vial. For deprotonation, 10 µl of 100% TCA containing 1 M Na_2_CO_3_ was added to the sample, vortexed and incubated on ice for 5 min. Afterwards, pH neutrality was determined using pH test strips. Zn standard dilutions were measured at room temperature at Ex/Em = 485/525 nm using the EnSpire™ Multimode Plate Reader (PerkinElmer Inc, USA) to establish a standard curve. 50 µl of sample was applied for quantification. Data from different plates were normalized according to the Zn standards.

### 2.6 Calcium quantification

Free Ca levels were quantified using the Calcium Assay Kit (Colorimetric) (ab102505, Abcam^®^, United Kingdom) following the instructions for tissue samples with slight modifications: Tissue samples were weighed and were suspended in 500 µl Calcium Assay Buffer (provided in the kit) in 1.5 ml screw-cap tubes containing glass beads. Tissue was disrupted as described for the RNA extraction method. The optical density (OD) of the Ca standard dilutions was determined at room temperature at 575 nm using the EnSpire™ Multimode Plate Reader (Perkin Elmer Inc, USA) to establish a standard curve. 50 µl of sample was applied for quantification. Data from different plates were normalized according to the Ca standards.

### 2.7 Protein extraction and western blots

For total protein extraction, tissue samples were homogenized in 2 ml Eppendorf tubes containing 500 µl lysis buffer [25% glycerol, 1.5 mM MgCl_2_, 0.2 mM EDTA Titriplex III, 40 mM Hepes, 430 mM NaCl_2_] including protease inhibitors [1 mM sodium vanadate, 0.5 mM PMSF, 1 µg/ml aprotinin, 1 µg/ml leupeptin, 1 µg/ml pepstatin, 0.5 mM DTT] and glass beads using the Precellys^®^ Evolution tissue homogenizer (Bertin Instruments, France) as follows: 5000 rpm, 2 x15 s with a 15 s pause in between, which was repeated after a 3 min pause on ice. The homogenates were centrifuged at 2348 rcf for 5 min at 4°C, the supernatant was transferred to a fresh tube, which was repeated once. Protein concentration was determined using a NanoDrop™ 2000 spectrophotometer (ThermoFisher Scientific, USA). 25 μg total protein for P-AMPK and 40 μg total protein for HSP70 were mixed 1:1 with 2x Laemmli buffer (Bio-Rad Laboratories, Inc., USA) and were heated at 95°C for 5 min. Samples were separated on a 12% Criterion TGX Stain-free precast gel (Bio-Rad Laboratories, Inc., USA) in a Criterion vertical electrophoresis cell (Bio-Rad Laboratories, Inc., USA) containing 1x Tris-glycin buffer for 90 min at 125 V. The PageRuler™ Prestained Protein Ladder (Thermo Fisher Scientific, USA) was used as a size standard. The gel was then UV activated for 1-2.5 min using a Chemidoc (Bio-Rad Laboratories Inc, USA) for total protein visualization. A PVDF blotting membrane (Bio-Rad Laboratories Inc, USA) was activated with methanol for 5 min and filter paper was soaked in 1x semidry buffer containing 20% Methanol. Then, the gel was blotted to the PVDF membrane for 30 min at 25 V using a Trans-Blot^®^ Turbo Transfer System (Bio-Rad Laboratories Inc, USA). After transfer, the membrane was washed with 1x TBS-T (Tris buffered saline with Tween) and was then added to 1x TBS-T containing 5% milk powder for 1 h at room temperature on a shaker to block the remaining binding sites. The membrane was incubated overnight at 4°C on a shaker in 1x TBS-T containing 5% milk powder and the primary antibody (rabbit anti-phospho-AMPKα [Thr172] from Merck KGaA (Product Number 07-626), Germany, diluted 1:1000; mouse monoclonal anti-heat shock protein 70 from SIGMA Aldrich (Product Number H 5147), USA, diluted 1:2500). The membrane was rinsed twice with 1x TBS-T, followed by three 10 min washes with 1x TBS-T. The membrane was then incubated for 1 h on a shaker at room temperature in 1x TBS-T containing 5% milk powder with the secondary antibody (goat anti-rabbit IgG H&L (HRP), Abcam, UK; anti-mouse IgG (whole molecule) – peroxidase antibody produced in rabbit, Sigma-Aldrich, USA, both diluted 1:10000). Detection was performed using Amersham™ ECL Select™ Western Blotting Detection Reagent (Cytiva, USA) in a Chemidoc (Bio-Rad Laboratories, Inc., USA). To test specificity of the antibodies, mouse protein from NIH 3T3 cells was used as a positive control to make sure that the correct band was chosen for analysis in earthworm samples (**Supplementary Figure S1**). Total protein was quantified from the stain-free precast gels and used as loading control for normalization. The positive controls and an example for a loading control are shown in **Supplementary Figure S1**. All membranes that were used for analysis are shown in the **Supplementary Material (Supplementary Figure S2**). All samples from one treatment group and time point were loaded onto the same gel together with the respective control samples.

### 2.8 Protein & carbohydrate quantification

Tissue samples were taken from individuals of each treatment group, stored at -80°C and homogenized in Millipore grade water using the Precellys^®^ Evolution Tissue Homogenizer (settings as in 2.7). 200 µl of homogenate was incubated with 30 µl 100% TCA for 10 min at -20°C. After centrifugation (3000 g, 10 min, 4°C) the supernatant was transferred to a new tube. The pellet was dissolved in 100 µl 5% TCA and centrifuged again under the same conditions. The supernatant was added to the first supernatant. The pellet was resuspended in 100 µl 1 M NaOH and the protein content was measured using a NanoDrop™ 2000 spectrophotometer (ThermoFisher Scientific, USA). Carbohydrates were determined according to a previous study (39) with the following specifications. D(+)-Glucose (Roth, Germany) was used as the standard. 150 µl H_2_SO_4_ and 30 µl 5% phenol were added directly to 50 µl supernatant and incubated at 90°C for 5 min. Measurements were accomplished at 490 nm using a Victor X4 plate reader (Perkin Elmer, Waltham, MA, USA), since most of the sugars can be detected at this wavelength.

### 2.9 Statistical analysis

Statistical analyses were performed using GraphPad Prism 9.5.1 software (GraphPad Software, San Diego, CA, USA). Technical outliers were identified and removed using the Grubbs test (p < 0.05). Normality was tested using the Kolmogorov-Smirnov test. For normally distributed data, ANOVA (including Bonferroni correction) or student’s t-test was performed. If the variance was significantly different (p < 0.05) Welch’s correction was performed. Kruskal-Wallace test (including Dunn`s test) or Mann-Whitney U test was calculated for non-normally distributed data. For comparing Tissue 1 and Tissue 2 samples mixed-effects analysis was performed in GraphPad Prism 9.5.1 software.

Principal component analysis (PCA) was performed in RStudio 2022.07.1 using the PCA script from the FactoMineR package. For visualization we used fviz_pca from the factoextra package. An ellipse with a confidence level of 0.95 was added. Correlation was accomplished using the cor script from the stats version 4.2.1. package. To overcome the issue of missing values for principal component and correlation analysis we used the mean values of each analysis, time point and treatment group.

## 3 Results and discussion

Many research disciplines consider earthworms as important model organisms, such as ecotoxicology and regenerative biology (40, 41). In the present study, we aimed to bridge both disciplines and investigated the effects of cadmium exposure, wound healing, and the combination of both stressors in the earthworm species *L. terrestris*. Moreover, we resolved the question whether detoxification and wound healing are cellular processes that interfere with each other, which has previously been proposed in a study showing that injury inhibits Cd-induced MT2 gene expression in *L. terrestris* and *L. rubellus* (23). However, the latter study did not distinguish between harmed and unharmed tissue, in contrast to the present study. Herein, we observed a similar effect in the anterior sections (Tissue 2), namely that Cd-induced MT2 expression was suppressed, but only in amputated earthworms.

**Figure 1:**
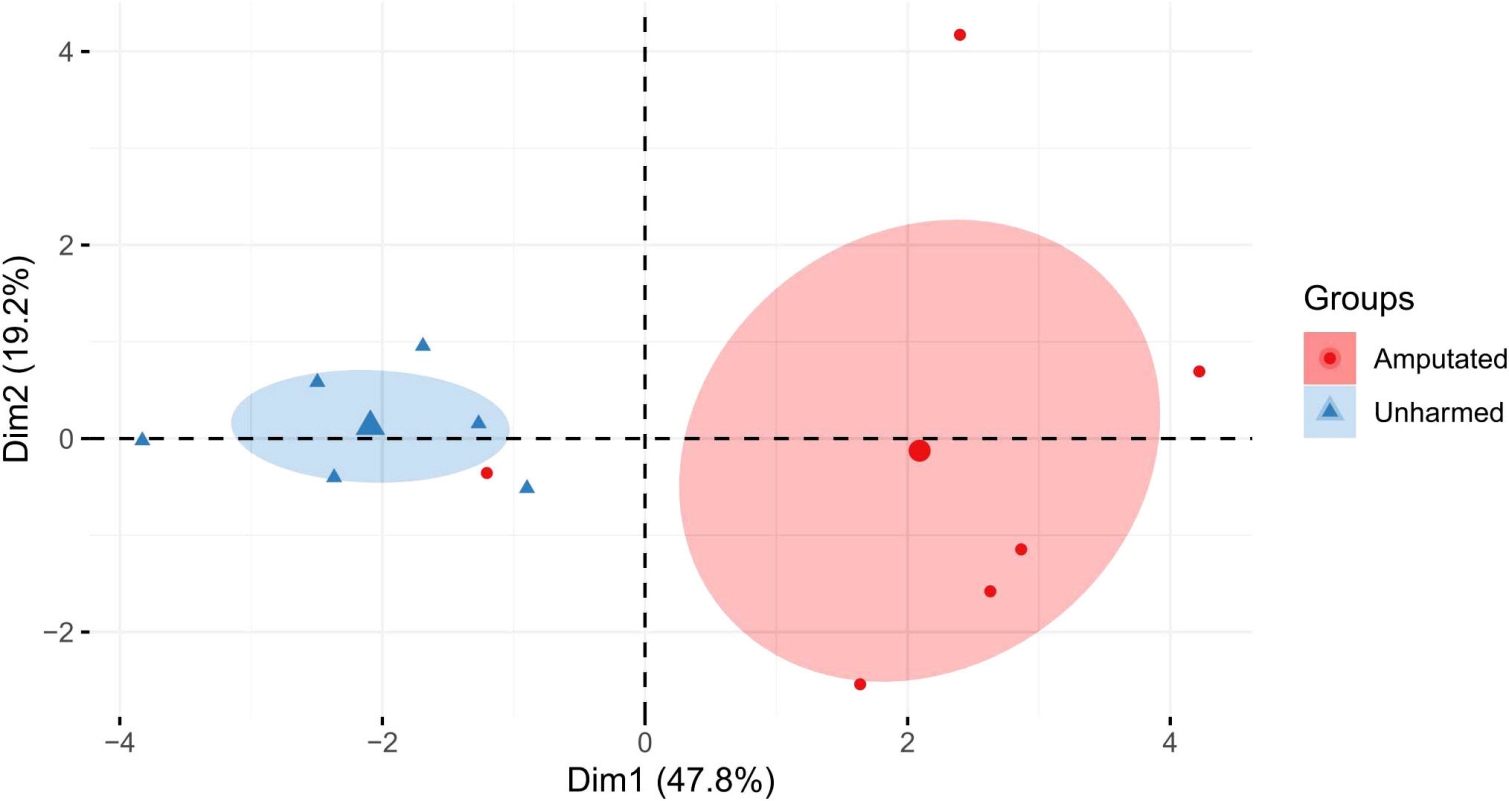
Principal component analysis (PCA) based on the mean values of each analysis from amputated and unharmed *L. terrestris.* The percentage of variation between the treatment groups for dimension 1 (Dim1) and dimension 2 (Dim2) are shown. The ellipse represents a confidence level of 0.95.

PCA revealed a clear separation of the treatment groups along Dim 1 (47.8%) by the health status of the earthworms (amputated or unharmed) rather than by Cd exposure (**Figure 1**). Except for a significantly increased MT2 gene expression level, which is known to be the major detoxification mechanism of Cd (14) at week one, significantly decreased HSP70 and P-AMPK levels, and a significant increase in P-AMPK after two weeks, no other exclusively Cd-related effects were detected. PCA showed that P-AMPK, HSP70, MT1, and MT2 were the main factors to separate the groups along Dim 2 (**Supplementary Table S4**), explaining 19.2% of the variation. The factors metal and time did not contribute to the group’s separation (**Supplementary Figure S3**). The results of the correlation analysis are shown in **Supplementary Figure S4**.

**Figure 2:**
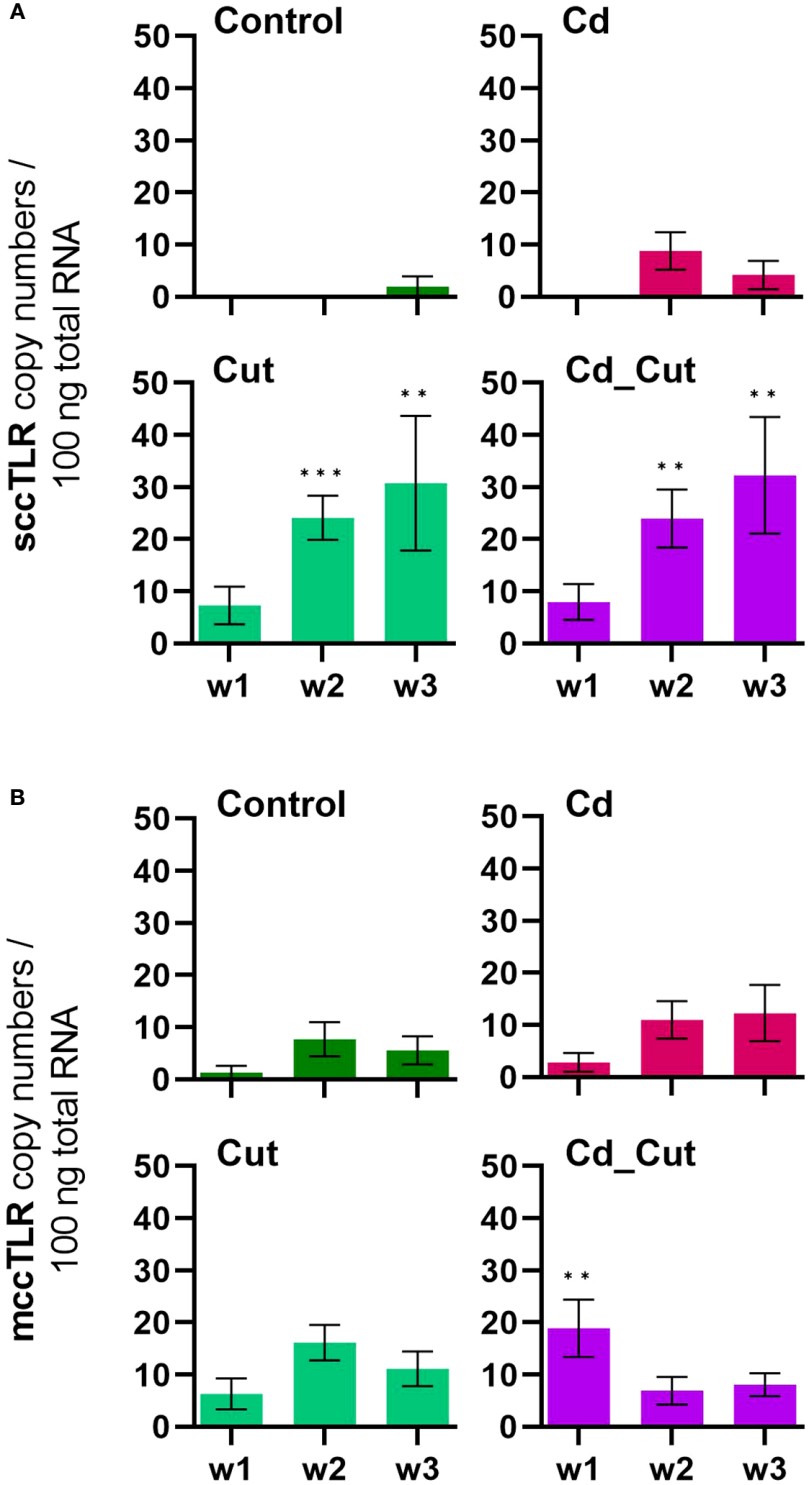
Absolute mRNA copy numbers of **(A)** single cysteine cluster toll-like receptor (sccTLR) and **(B)** multiple cysteine cluster toll-like receptor (mccTLR) in *L. terrestris* tissue from Control and exposed (50 mg CdCl_2_/kg dry soil (Cd)) individuals; regenerative tissue of amputated (Cut) and exposed amputated [50 mg CdCl_2_/kg dry soil (Cd_Cut)] individuals at week one (w1), week two (w2), and week three (w3). Asterisks indicate significant differences between treatments and the control of the respective week [p < 0.01 (**); p < 0.001 (***)]. Mean values ± SEM are presented.

**Figure 3:**
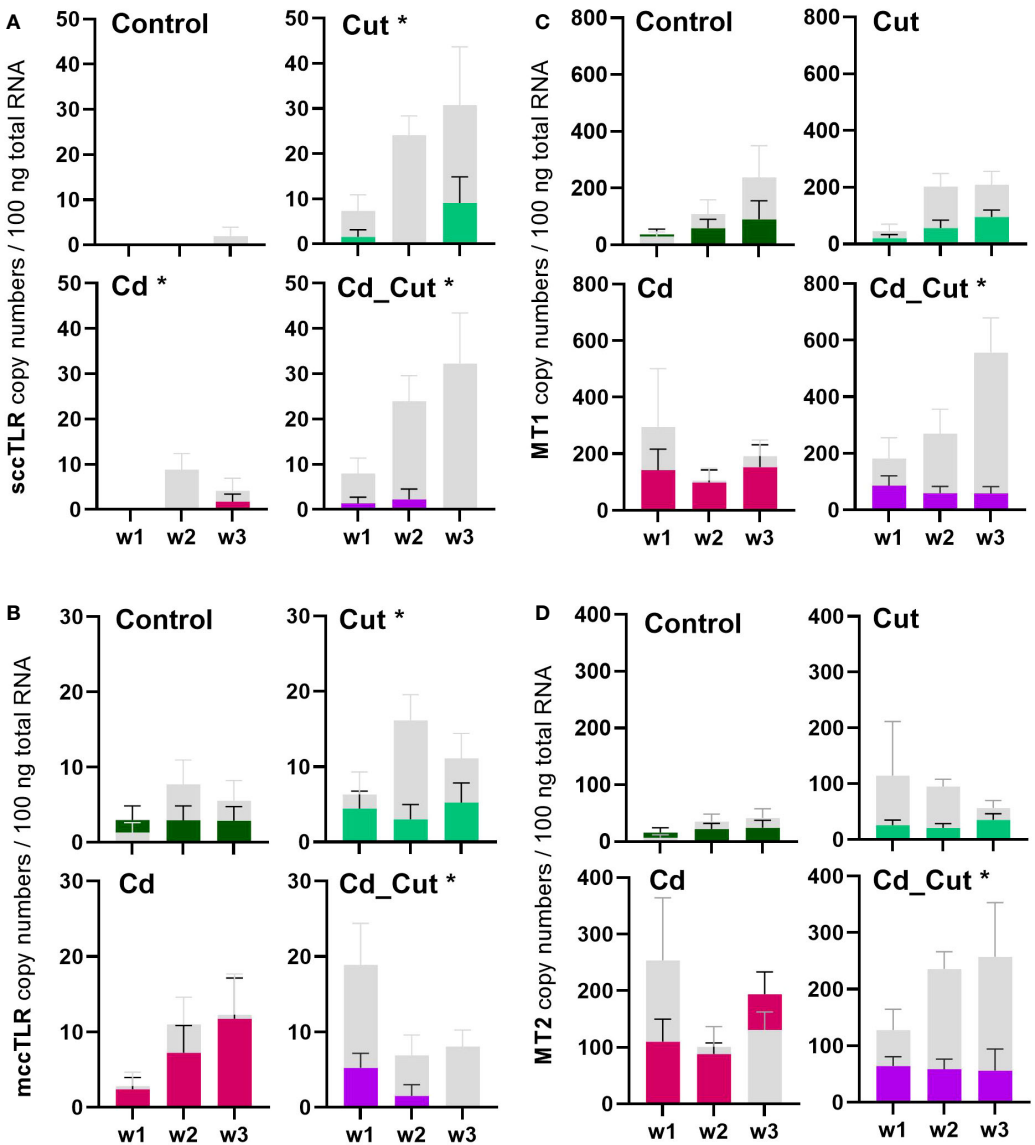
Comparison of absolute mRNA copy numbers in Tissue 1 and Tissue 2 of **(A)** single cysteine cluster toll-like receptor (sccTLR); **(B)** multiple cysteine cluster toll-like receptor (mccTLR); **(C)** metallothionein 1 (MT1) and **(D)** metallothionein 2 (MT2) in *L. terrestris* tissue from Control; exposed [50 mg CdCl_2_/kg dry soil (Cd)]; amputated (Cut) and exposed amputated [50 mg CdCl_2_/kg dry soil (Cd_Cut)] individuals at week one (w1), week two (w2), and week three (w3). Grey bars represent gene expression in Tissue 1 (including regenerative tissue) and colored bars represent gene expression in Tissue 2 (anterior section, no regenerative tissue). Asterisks indicate significant differences between Tissue 1 and Tissue 2 using mixed-effects analysis [p < 0.05 (*)]. Mean values ± SEM are presented.

Cd is generally known to be an immunotoxic agent (8). In a previous study, we were able to show that earthworm immune cells – coelomocytes - are affected regarding their metabolism (42), but also chromosomal aberrations and single-strand breaks have been observed upon Cd exposure (43). In earthworms, a dose-dependent change in the expression of antimicrobial peptides by Cd (44) has been shown, as well as changes in lysenin and coelomic cytolytic factor 1 (CCF-1) levels, two factors which are important in immune defense (45). A study on the blue mussel showed that Cd led to the upregulation of several TLRs (46). We identified two partial *L. terrestris* TLR sequences (**Supplementary Table S3**). Both, sccTLR and mccTLR, did not reveal Cd-dependent changes. SMART™ protein domain analysis revealed one sccTLR containing an intracellular Toll -interleukin 1-resistance (TIR) domain (141 aa), a transmembrane region (22 aa), a single leucine rich repeat C-terminal (LRR_CT) domain (54 aa) and six internal extracellular leucine-rich repeats (LRRs) (23-24 aa) (**Supplementary Figure S5A**). The second TLR was identified as a mccTLR, containing an intracellular TIR domain (144 aa), a transmembrane region (22 aa), two LRR_CT domains (48 aa and 60 aa) with two LRR-C-terminal domains (22 and 23 aa) and one LRR-N-terminal domain between them (38 aa), followed by 12 internal extracellular LRRs (19 - 23 aa) (**Supplementary Figure S5C**). **Supplementary Figures S5B**, **S5D** shows the comparison to the *E. andrei* TLRs. We observed a significant induction of sccTLR in amputated- in contrast to Cd-exposed earthworms (**Figure 2A**). Interestingly, we could hardly observe any gene expression of sccTLR in Tissue 2 (**Figure 3A**). On the other hand, mccTLR revealed constitutive gene expression in both tissue sections (**Figure 3B**) and a significant difference between the control and Cd_Cut group at week 1 (**Figure 2B**). Therefore, the time and dose of Cd exposure might play an important role regarding the change of immunity-related genes.

**Figure 4:**
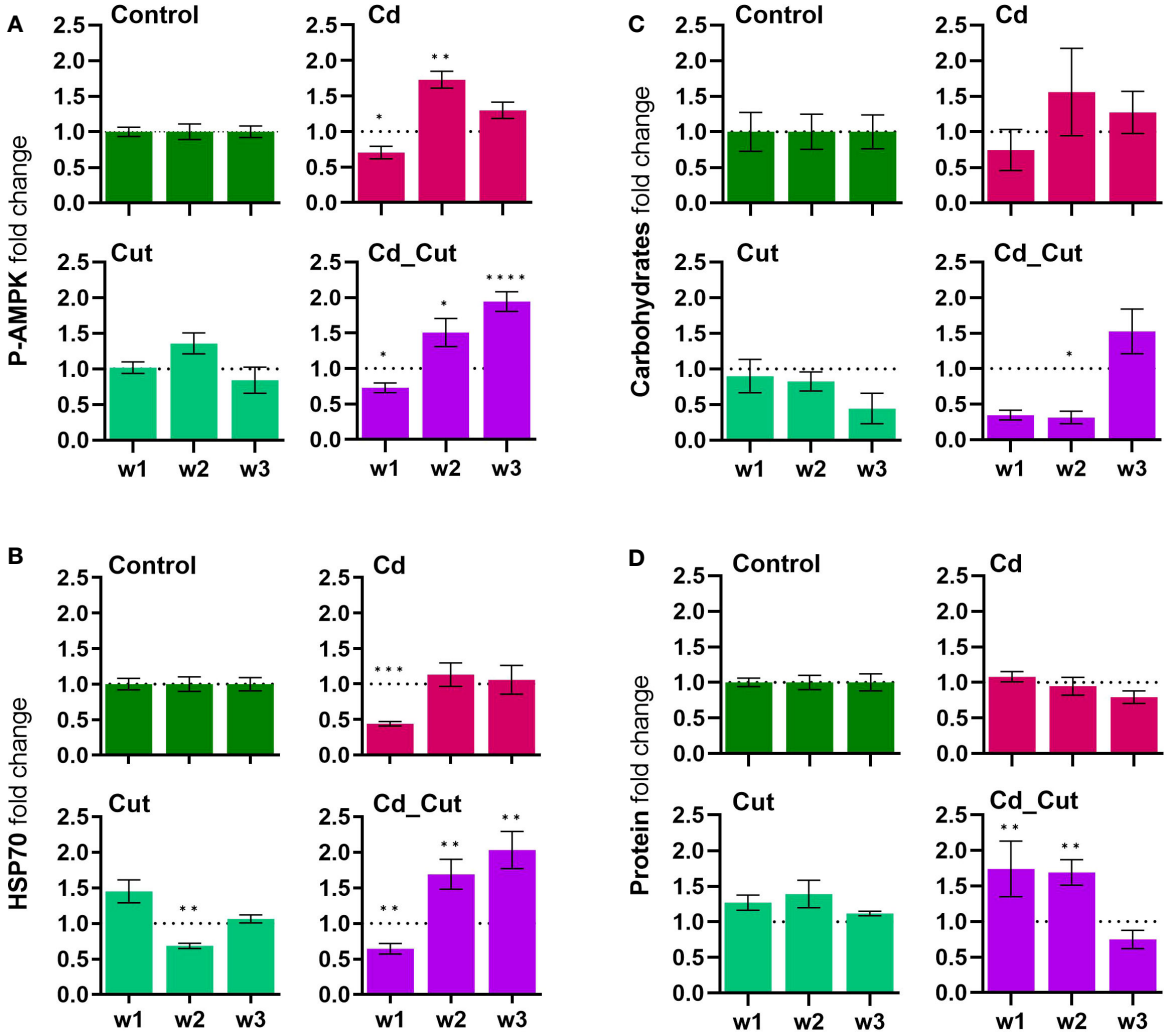
Comparison of fold change normalized to control of **(A)** phosphorylated AMP-activated protein kinase (P-AMPK); **(B)** heat shock protein-70 (HSP70); **(C)** total carbohydrates; **(D)** total protein in *L. terrestris* tissue from Control and exposed [50 mg CdCl_2_/kg dry soil (Cd)] individuals; regenerative tissue of amputated (Cut) and exposed amputated [50 mg CdCl_2_/kg dry soil (Cd_Cut)] individuals at week one (w1), week two (w2), and week three (w3). Asterisks indicate significant differences between treatments and the control of the respective week [p < 0.05 (*); p < 0.01 (**); p < 0.001 (***); p < 0.0001 (****)]. Mean values ± SEM error are presented.

P-AMPK decreased after one week of Cd exposure, indicating that sufficient energy is on hand. This was followed by an increase in all treatment groups after two weeks, P-AMPK peaked after three weeks in the combined stress group, which hints towards low ATP levels and an elevated energy need (**Figure 4A**). It seemed that Cd had no effect on the energy metabolism. Interestingly, carbohydrate and protein levels showed a quite strong negative correlation (**Supplementary Figure S4**). Only the combined treatment group showed significant changes in carbohydrate and protein levels. In the Cd_Cut group, after one and two weeks, carbohydrate levels were low and protein levels were high, whereas after three weeks it was the opposite – we observed lower protein and higher carbohydrate levels (**Figures 4C, D**).

Cd exposure indeed triggered a carbohydrate depletion after 8 days in study on *E. albidus* (34). In the present study, only the combined stress group revealed an increased energy demand and a depletion of carbohydrates at first. Interestingly, the respective protein levels showed an opposite effect. An important role of lipids cannot be excluded, which we, however did not determine herein. The situation changed after three weeks – carbohydrate levels seemed to recover while protein levels decreased. P-AMPK levels remained elevated after three weeks of combined exposure suggesting that only amputated earthworms exposed to Cd still had an increased energy need.

A previous study revealed that AMPK negatively regulates HSP70 expression in HepG2 cells and that Cd induces HSP70 expression (47). After one week, the most obvious result herein was that Cd caused a downregulation of HSP70. After two weeks and most obviously after three weeks, HSP70 levels were significantly increased in the combined stress group (**Figure 4B**).

The heat shock response is a protective mechanism under environmental stress that is involved in protein folding, degradation, etc. We observed a similar expression pattern between P-AMPK and HSP70 levels – if these two proteins are linked at a regulatory level remains to be elucidated. Recently, a review article revealed that Cd leads to an upregulation of HSP70 (48), however, there is also evidence that Cd decreases HSP70 levels like for example in Cyprinidae fish (49) and zebrafish (50). The latter study revealed that the Cd-induced decrease in HSP70 could be reversed by pre-exposure of animals to Zn. We were not able to link Zn levels to the expression of HSP70 in the tissues of the earthworm *L. terrestris*. Earthworms exposed to Cd for three days showed increased HSP70 expression in coelomocytes (51) as well as sea star coelomocytes six hours post-traumatic stress (52). Extracellular HSP70 has been earlier shown to regulate innate immune cell function (53). Arsenite exposure induced the expression of HSP70, which subsequently reduced the anti-inflammatory response to lipopolysaccharides in canine 030D cells (54). It would be interesting to evaluate a putative link between HSP70 and the anti-inflammatory response in earthworms in future studies.

**Figure 5:**
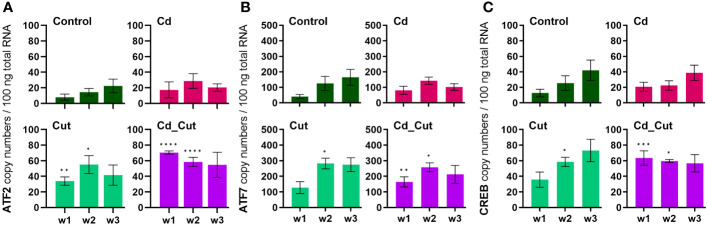
Absolute mRNA copy numbers of **(A)** activating transcription factor 2 (ATF2), **(B)** activating transcription factor 7 (ATF7) and **(C)** cAMP response element-binding protein (CREB) in *L. terrestris* tissue from Control and exposed [50 mg CdCl_2_/kg dry soil (Cd)] individuals; regenerative tissue of amputated (Cut) and exposed amputated [50 mg CdCl_2_/kg dry soil (Cd_Cut)] individuals at week one (w1), week two (w2), and week three (w3). Asterisks indicate significant differences between treatments and the control of the respective week [p < 0.05 (*); p < 0.01 (**); p < 0.001 (***); p < 0.0001 (****)]. Mean values ± SEM are presented.

We identified partial sequences of ATF2, ATF7 and CREB (**Supplementary Table S3**). These factors contributed the most to the separation of amputated earthworms (Cut and Cd_Cut) along Dim1 (**Supplementary Table S4**). ATF2 copy numbers revealed a significant increase in amputated earthworms after 1 and 2 weeks (**Figure 5A**). ATF7 gene expression increased significantly after two weeks in amputated earthworms (Control and Cd-exposed), and showed an additional induction in the Cd_Cut group after one week. CREB showed the same pattern (**Figures 5B, C**). A comparison of ATF2, ATF7 and CREB between Tissue 1 and Tissue 2 is presented in **Supplementary Figure S6**. It has recently been described in mice that ATF2 and ATF7 protect the intestinal epithelium from apoptosis during regeneration (55), whereas CREB is known to regulate cell proliferation and differentiation (30, 56, 57). Both factors revealed a strong positive correlation to sccTLR. Scc- and mccTLRs have been previously described for the earthworm species *E. andrei* (33), the sccTLR (EaTLR) was elevated in response to bacterial stress (32), while another study revealed that EaTLR showed a decreased expression anterior- but no changes in posterior regeneration (22). Moreover, mccTLRs were shown to be expressed in sea urchin coelomocytes after bacterial challenges (58). It is known from the literature that TLRs directly interact with ATF family members, such as ATF4 and TLR4 leading to cytokine production (59). In addition, several TLRs activate ATF2 leading to the stimulation of genes related to cellular motility (31) and that ATF2 protein is highly expressed in infiltrating murine macrophages in adipose tissue (60). Since we could show that sccTLR is increased in regenerative tissue, we suggest that sccTLR signaling recruits ATF7 and CREB in earthworms. mccTLR, in contrast to sccTLR, was hardly induced in regenerative tissue and did not show the same strong positive correlation to ATF7 and CREB, suggesting that mccTLR has a different function, probably not directly related to wound healing.

**Figure 6:**
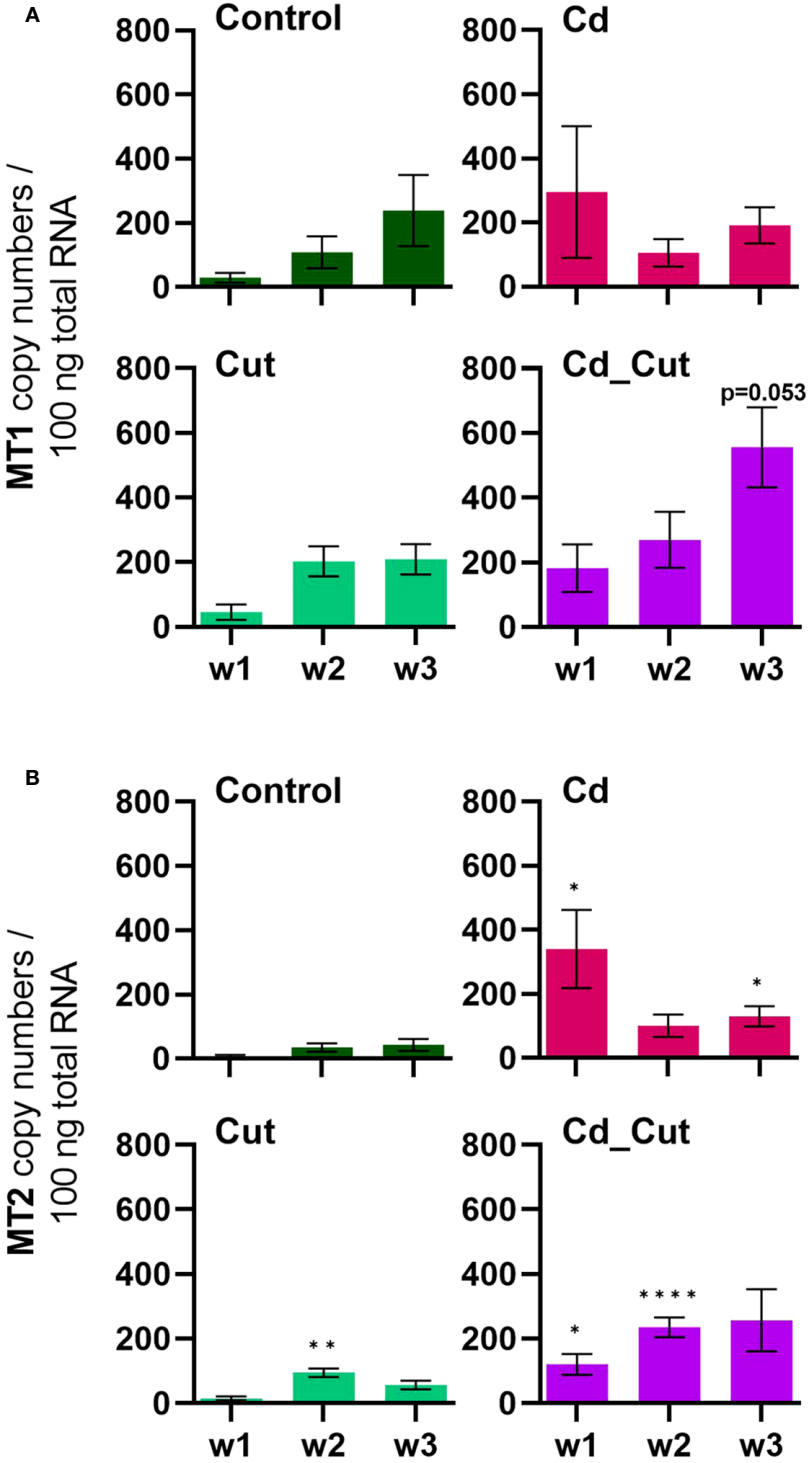
Absolute mRNA copy numbers of **(A)** metallothionein 1 (MT1) and **(B)** metallothionein 2 (MT2) in *L. terrestris* tissue from Control and exposed [50 mg CdCl_2_/kg dry soil (Cd)] individuals; regenerative tissue of amputated (Cut) and exposed amputated [50 mg CdCl_2_/kg dry soil (Cd_Cut)] individuals at week one (w1), week two (w2), and week three (w3). Asterisks indicate significant differences between treatments and the control of the respective week [p < 0.05 (*); p < 0.01 (**); p < 0.0001 (****)]. Mean values ± SEM are presented.

Previously, ATF family members were hypothesized to play a role in Cd-induced MT2 gene expression (23). Since we did not observe a correlation between ATF2, ATF7 or CREB and MT2 gene activity, we suggest that these factors are not involved in MT2 gene regulation. A study on Cd-exposed *E. andrei* proposed for MT2 not only a role in Cd detoxification, but also a protective role during regeneration since in amputated and Cd-exposed earthworms MT2 expression was increased and regeneration was even faster in the presence of Cd (61). If wound healing in *L. terrestris* is accelerated in the presence of Cd, has to be addressed in future studies. MT2 expression increased in earthworms upon exposure to Cd (**Figure 6B**). However, a Cd-induced induction of the MT2 gene failed in Tissue 2 of amputated earthworms as shown in the Cd_Cut group (**Figure 3D**).

**Figure 7:**
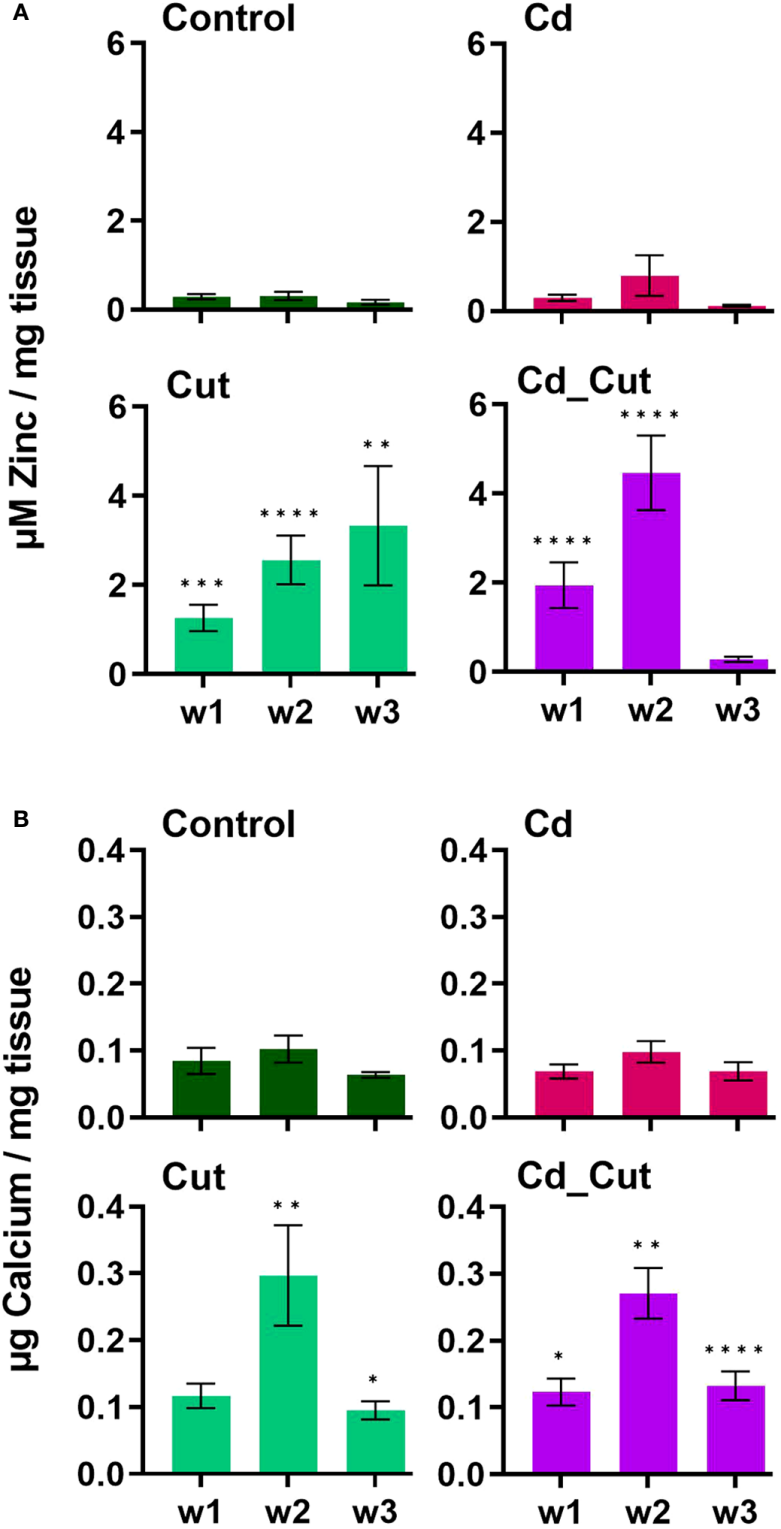
Levels of **(A)** total zinc and **(B)** free calcium in *L. terrestris* tissue from Control and exposed [50 mg CdCl_2_/kg dry soil (Cd)] individuals; regenerative tissue of amputated (Cut) and exposed amputated [50 mg CdCl_2_/kg dry soil (Cd_Cut)] individuals at week one (w1), week two (w2), and week three (w3). Asterisks indicate significant differences between treatments and the control of the respective week [p < 0.05 (*); p < 0.01 (**); p < 0.001 (***); p < 0.0001(****)]. Mean values ± SEM are presented.

We identified a partial *L. terrestris* MT1 sequence (**Supplementary Table S3**). The highest induction of MT1 was observed in the Cd_Cut group after three weeks (p = 0.053) but only in Tissue 1 (**Figure 3C**). The Cd and Cut groups did not show significant differences from the respective control (**Figure 6A**). Numerous immunomodulatory functions have been suggested for MTs in humans, such as the role of MT1 as an enhancer in cells of innate immunity (62). Few functional studies on earthworm MT1 have been accomplished so far. A role for mt1 in *Dendrobaena octaedra* has been suggested in copper resistance but only in adapted populations (63). A combined treatment of copper exposure and freezing also lead to an upregulation of mt1 in *D. octaedra* (64). The involvement of *L. rubellus* MT1 in metal homeostasis, e.g. as a donor for essential metals like Zn, was proposed several years ago (13). Zn is required at all stages of wound healing (65) and it is essential for several functions in innate immunity (66). Zn is also known to be relevant for TLR/nuclear factor k-light-chain-enhancer of activated B cells (NF-kB) mediated inflammatory signaling and cytokine production (65). Total Zn and free Ca have been measured. Both, Zn and Ca levels increased significantly in amputated earthworms (Control and Cd-exposed), whereas Cd alone did not lead to significant changes in either Zn or Ca levels (**Figures 7A, B**). Interestingly, Zn levels were the highest in the combined stress group after two weeks, but dropped to control levels after three weeks. Interestingly, at the same time, MT1 levels were increased. Since we measured the total amount of Zn, we can exclude a role for MT1 in Zn homeostasis. Taken together, we propose a function of MT1 in the response to multiple stressors and a putative contribution to stress adaptation.

A previous study showed that short-term exposure to Cd increased Ca levels in *L. terrestris* coelomocytes (42), which is known to be necessary for coelomocyte activation (67). Injuries in *Drosophila* embryos and zebrafish larvae showed that the first signal in the wound inflammatory response is Ca flashes leading to the release of hydrogen peroxide, which acts as an attractant for immune cells that are relocated to the site of injury within minutes. Moreover, Ca is known to regulate cell differentiation, contraction to reduce the wound size, and angiogenesis, and remains elevated during the inflammatory and proliferative phases while Ca levels decrease during the remodeling phase (68). Herein, we measured free Ca in tissue samples revealing that Ca levels peaked after two weeks and declined in week three, although levels were still significantly increased in amputated earthworms compared to controls. Considering human models, we conclude that earthworms transition from the proliferative into the remodeling phase between week two and three.

## 4 Conclusion

Earthworms are well prepared for environmental stress due to their soil-dwelling lifestyle, which entails different challenges ranging from physical stress, soil pathogens to xenobiotics. Mainly, these challenges do not occur as single events, but as a combination of stressors. We were able to show that the combination of stressors had the most severe effect. We also revealed spatial expression of MT2 and sccTLR along the posterior part of the earthworm. We think that the difference in gene expression between Tissue 1 and Tissue 2 is due to the distribution of coelomocytes during wound healing. In a previous study, we showed that MT2 expression is higher in coelomocytes than in tissue samples (9) and it is also known that TLRs are expressed in coelomocytes (58). Coelomocytes infiltrate regenerating tissue and therefore relocate to the wound site, which could explain the differential gene expression levels of MT2 and sccTLR in Tissue 1 compared to Tissue 2. However, the reason why we still don’t observe Cd-induced MT2 induction in Tissue 2 after three weeks, when the numbers of coelomocyte should have recovered, still needs to be clarified. We can only speculate that a combination of stressors slows down proliferation of coelomocytes, which might be reinforced by a challenged energy metabolism.

## Data availability statement

The original contributions presented in the study are publicly available. This data can be found here: https://www.ncbi.nlm.nih.gov/nuccore; OR596220-OR596222, OR610157-OR610159.

## Ethics statement

Ethical approval was not required for the study involving animals in accordance with the local legislation and institutional requirements because there is no ethical approval required for invertebrate species.

## Author contributions

MH: Conceptualization, Data curation, Formal Analysis, Funding acquisition, Investigation, Methodology, Project administration, Resources, Software, Supervision, Validation, Visualization, Writing – original draft, Writing – review & editing. GA: Data curation, Formal Analysis, Investigation, Methodology, Supervision, Validation, Visualization, Writing – original draft, Writing – review & editing. VP: Data curation, Investigation, Methodology, Visualization, Writing – review & editing. BF: Data curation, Methodology, Writing – original draft. CP: Writing – review & editing.
