# Supplementary material for: Wound healing and Cadmium detoxification in the earthworm Lumbricus terrestris – a potential case for coelomocytes?
Source: Front Immunol. 2023 Dec 5;14:1272191. doi: 10.3389/fimmu.2023.1272191 (PMC10728717; doi:10.3389/fimmu.2023.1272191)
Supplement: Supplementary file 1 [file DataSheet_1.pdf]

Supplementary Material

Wound healing and Cadmium detoxification in the earthworm *Lumbricus terrestris* – a potential case for coelomocytes?

1 Supplementary Tables

1.1 Table S1. Number of biological replicates

Table S1. The number used in each assay is presented by week of collection and experimental group

Assay	Week	C	Cut	Cd	Cd_cut
**qPCR** (MT1, MT2, sccTLR, mccTLR, ATF2, ATF7, CREB)	1	6	7	7	7
	2	7	7	7	7
	3	7	7	7	7
**Western Blot** (P-AMPK, HSP70)	1	7	7	7	7
	2	7	7	7	7
	3	7	7 (5 for HSP70)	6	7
**Total Zinc quantification**	1	10	9	10	10
	2	10	10	7	10
	3	10	10	10	10
**Free Calcium quantification**	1	10	10	10	10
	2	10	10	10	10
	3	10	10	10	10
**Protein & carbohydrate quantification**	1	6	6	6	6
	2	5	6	5	5
	3	6	6	5	6

1.2 Table S2. Primers used for qPCR

Table S2. Primers used for qPCR are listed with the genes to amplify and their sequences.

**Gene**	**Forward primer (5′ - 3′)**	**Reverse primer (5′ - 3′)**
**MT1**	CGTGTCCAAGGGAAGGATCA	GAATGCCTGCCAAATTGTGA
**MT2**	TGCAGGTGTCCAAAAGATGA	ATCAGCACAGCAAAGCTTCTTG
**sccTLR**	CGTGACAACCTCATTCTGGTGAT	TGCAACATGAATCCCAGGC
**mccTLR**	ATCAACGCCGGTGATCTGA	CTAATGGTAGACGTCACGGCC
ATF2	CTGGAGCACGCTCTGTTGAA	AAGACGCTGCTTTGTTGTGCT
**ATF7**	GCGGACCTCACCCCAACT	AGTTGCGATGAGGTTGGGC
**CREB**	TGAGGAGTTAAAGTCGCTGAAAGA	GCATAGCTAGCCTGCACGC

1.3 Table S3. Nucleotide sequence of MT1, sccTLR, mccTLR, ATF2, ATF7, and CREB

Table S3. MT1, sccTLR, mccTLR, ATF2, ATF7 and CREB nucleotide sequences (5’-3’) from *Lumbricus terrestris*. Amplicon sequences are highlighted.

GENE	**Newly derived sequences (5´-3´) of *Lumbricus terrestris***
**MT1**	TGGCTGATGCAGGACCGTGCAATACTAAATGCTGTGGAAAGCCCTCGTGTCCAAGGGAAGGATCAAAGTGCGTTTGCATTAAC<br>TGCAAATGTGAGAAGGGTGAATGCCTGCCAAATTGTGATAAGAATTGCTGTGGTGGAACGGAGCAGTGTGCTTCAAAATGTGG<br>AAATCCAAACTGCAAGTGTGGGGCTGACTGCAAGTGCGCACCTGGTCAATGTTCCACAGAGTGTGCGAAGGGATGTTGTGACT<br>AG
**sccTLR**	CGTGATAATAAGCTCTCAGTTGTCAACATAACAACTCTTCCGGAAACAATCTGGAATCGTCTAACCCGAGTCGATTTTTCCGAG<br>AACACTTTGTACTGCGATTGCCAAATTGTTTGGTTTCGTCGCTGGCTCAACAAAAAGAAGAACACGACCGTAGAAAACCTGAA<br>TCTGACTCGATGCAGTGGACCAGCAGAGGTGAAAGATGTACCAATACATCGTCTCAAGCATCCAACTGACTTGGAATGCTTCA<br>GTGAAGAGCAAGACGCCTGTCTTCTTTCTGTCTTTCTTACAGTTGTCCTCATTGTTTTGGTGTCATTTCTGGTCGCCATTCTACAC<br>AGACTGAGGTGGTTTCTAAAATACTGGTACTTCAGATACAAGGCAAGAGCGAAAGAGTTTCGAGAACTGGTTGATCATCACGA<br>TTACGAGTTCGACGCCTTCATCAGCTACAGCGARACAAAYTACGAATGGATCCTCGACCAGCTTCATCCACGACTTGAAAAYG<br>AGTTCGGTCTCCACTTRTGCATCCACCACAGAGACTGGTCGCTGGGRCGAGACATCGTTGAGAACATAGTGAACAGCATCGCG<br>CACAGTCGTAAGACGGTGTTGATCGTGTCAAACGCCTTCGCCGTGTCGCAGTGGTGTCACTTCGAAATGACGATGGCCCAGAC<br>GAAGCTGTTCGAAGACGATCGTGACAACCTCATTCTGGTGATGCTGGAGGAGATCGCCGACTGCAACATGAATCCCAGGCTTC<br>AGCTGCAGATGCAGAAGAAGACGTACATCGARTGGACGGACAACGAGATCGGTCAGCAGTTGTTCTGGGAGAAGCTGAGACA<br>GGCTTTGGTCAAACCGTCGGAAAGTCTCATCAATGAAACTCCCCCGAGACAACTATTTGCTTGAAGAAGATAGTGGCGGAGCC<br>AGGATTTCAGTTTTGGGGACTGAAGCCTCGCGGAGGAAATCTTCAATTTTCATCCGCCTAAATTTCTGATGACCTTTTTTAGTC<br>ATCCGGCCGAAATCGTG
**mccTLR**	CTAAACCTGACCGCCGTGGACGTCAACAAATTCCTGACCAACGAATCAGAAAGTCTGAGACTGTTGGTATTGGCGCAAAACAA<br>CCTGTATTCGATAGAAGAGAATGCTTTTGAAGACCTACCGACTTCACTTAAGGAAGTCGATTTATCGCGGAATAATATTCGCCA<br>GCTGTCCTGGAAAACCTTTTCCAAACTGGAGAGCGTCAACATTTCCTGCAACCTACTGCGCTCCGTTGACTGCGGAGGAGGTC<br>AACAAGGACATTGTGGCGTCAACAACAGCCTTCGAATCTTAGATCTCAGCGACAACCTGCTGACCGAGATCCCGATCCTCAAA<br>GAACATCTCATAGCGATGCCAAACCTTAGCGTTCTCAACGTCTCTAAGAACAGACTTGACAGGCTGACTGTTCTTCCGGGAAG<br>TCTGGTGGAGTTGCGGGCCAGCGAGAACGAGCTCACAGCCATCGAAAAGGGAATCTTCTCATCACGATTGGAACAATTGCAG<br>ATTATTGATCTGTCTCGTAACAAACTCAATGCGATTATCAACCACACATTCGTTGACATCGGATCCATTCGGATGATCAATCTT<br>TCGCACAACGCAATCGAAACCATTGAGTCGTCGGCGTTCTACTCCACCCCTCCACGGATTCGATCCGGCGTGAAGCTGGAAGA<br>GTTCAGAGATGGAGATGAGAGCGACGTAGCGAATCCATGTGAGGGTAAGACCTGGTCCCAGCTGGAGATCGTCGATCTGAGC<br>CACAACGAACTGCGACATATAGACGATGCGGTGTTCATGGGGTCGCTGTGTGGCGTCAGGGAACTGCTGCTCGGATACAACCG<br>GATAGGGCGACTCTCGAGGTACATCTTGGAACAGCTGAGGCGCCTCGAGATCCTTGACCTTCAGCACAACGAACTGGCTTGGT<br>TGGAGGTGGGAACGTTCACGAGTCCCAGCCTGCGTCACATTGATCTGTCGCACAACGGTCTGCGGAAGATCATCAGCATGACG<br>TTCCTCTACCTTCCCACCGTCACCGACATCGACCTCAGCTACAACGAGATCGGATACCTGTACAGGTATGCCTTCTACCGGATC<br>TGCAAGGACTCTAACCAGCGCATCCGCATCTCGTTGCGTGGCAACCGGCTGCAGACGGATGCCGTCTGGAAGATGCTCTCTCT<br>GCTGCAGCACCAGGAGAACAGCACGTGCGCCATCGAAGCTGACCTACGTGACAACCAGGTGACGCACCTGATGGGCATTGAA<br>GCGATTCAGACGCTGAAGGCCCACATCAACGCCGGTGATCTGACGCGCTTTGCATTCTGGGAGCGTCTAATGGTAGACGTCAC<br>GGCCAACCCAGTGGAGTGCGACTGTCAGCTGACGGACGAACTGAACGCCATCTCGTACATCACCGGGACCTTCAACTCCTCGT<br>CGGGAAGGAAGAGTTTCGCCGCGTGGACGAGACTCAACTGCTCCCAGCCACCAGTCTTCCAGGGATTCAGCGTCTCCGAGTTC<br>CTCGCCTACTCGATCTGCCCCGTTCAGATCAGCTACGCTTCGTGTCCCAACGTCTGCGACTGCAGATTCGATATCCGCGGAAAG<br>GGAAACCGCTACATCAACTGCTCGCGGCGACACCTTGCGGAGTTTCCGGATTTCGAGTCGATTTACTTTAACGCAGTTGATCTA<br>TCGTACAACGACCTAACGGAAGTTCCGTCGGACTATTTCCGTTCCGCTGGTCACGTGGTCTACCTTGACCTCTCGCACAATCAT<br>CTGAAGACACTTCCGGGCGATGCACTGTTGCAGCTGACCTCCTTGAAGCACCTCTTCCTGCACCAGAACGACATTCGCTATCTC<br>CCGGCCGAGATCTCTCGTCTGCCGCAGCTCAAGTTCCTCAGCATCCAGGGTAACCCATTGTCTTGCGATTGCCGTCACGCCTCG<br>CTGAATCTCGTCTCTACAAAACTTCGCGGTGCAGTCTCTGACTTCCGACACGTGCGCTGCGCCGATGGTCGCTACCTGTGGCGA<br>CTGAACCTCACCCAGACGCTCGACTGCACTCTGGGTCTAACTAATACCCTGAGCGAAAATGGTCTCATGTCGCGGGTCCATGG<br>GGTACTGCTCGCCGTGTGCGTCCTAGTTGTGACCGGTGCTGTCGCTGTGCTTATCCTGAGGCGACTGAGAGCCAGTGCATCGCG<br>TGGATCGAGGGGGACGTGGCGAAAGATCGAGAACCAGGATGACCTCATCAAGCAGGACGTCTTCATTTCGTACAGCGATTCA<br>GACGAGGCTTGGGTGACGGGAACCCTTCTGGAGATCATCTGGAACGAGTCGAGTCAGTACAGCGTCTGTCTCCAGATGCAGCA<br>GCAGCAGCTGGAAGACTACGGGTCCCTCGGTCTCCACCTGGAGTCGGACACCATCCGGAGACACATCGACAACAGCAAGGTC<br>ACGATCGTCGTCCTTTCCACCCCGTTCCTGAAGAACGTCTGGGCGGTCAAGTCCTACCGCTCGACGGTCCTCGAGAAGCTCGAA<br>TCTCAGCGACACCAGTTCATCTTCGTCTACCTGGAGGCGGTGGACGTGGAGGAACAGGACCGTGACCTCCACGGATTCCTTCC<br>AGAGGACAAGACCCTGAAGGTGGCCGATCCTCTGTTCGCTGATAAACTCGTCTACCATCTTCCTCCGCCGTTGAAGACATCGC<br>GGAGCAGCCCCGCCAGCGGCAGCGGCAACCCCTCCGATCCGTGCAGCAGAGGACTTCTCGTTGAACTAACGGATGAAATCTCG<br>CCAGAGTACCACAGACAGAAATACTGATTGACTCGAACGGAGGTACTGACTGTCRTCGATGCTRACATACTATCTTGCGAART<br>CCTTCATGAATAGTACTTCTGTGGAGGATTCCAATGAGTTTTCAYACATTCCGATGGACATTTATACTCCTCKTCCACRATCGT<br>ATCGYKATTCCACGT

Supplementary Material

A<br>T<br>F<br>2	CACGATGACGGGGGGTCCACGGGCCACATGGCCCCGCGATGCCATTGTGCTCAGCGCGGCCATTGCTATCTCTTCGGCGTGCG<br>GCGTTATCATGATGGGGCTGCTGCTCGTCACGTCGTCCGACTGCATCTCAGTCTGAGCTGCTGCTGATGATGCACCCTGCAGTG<br>CCCTCTGTCGTCGTGTTACGGGACAGTCCTTGTGCGCCAGGAGCATCGTCCGTAACTGGACCACTTCGCTACGCAACTGAGTAA<br>CCTCCACCTCTAGCCTGCTATTGGCAGTTTGCAAATCTTCTGCTTTCAGCTCCAAACTGTTTACCCAAACCTTCTTCTTCTGTCG<br>ACATCTAGCGGCGGCAGCTCGATTTCGTTCTAAGAACTTTTTCCTTCTTTCGTCGGGATCGTCGTCTGTCGACAGTCGAGGTTTT<br>TTCGGCGTTGACGTATCCGCATCCTGGAGCTGAACCTCCGTTGACGACAGCTTGACTAGACGCGTCGTCGTCGTTGTCTCCGAA<br>GAAGCAGCGGTCGGCAGAAGCGACACGGGTCGAGGTGGCGTGGGTCGAGAGGCACCGCTAGAAGACGCCTGCTGGAGTTGG<br>GTCGTGACAAGGTCGACCGCATCCGACATGACCTGAATGTTGTTCTGGAGCACGCTCTGTTGAACGTTCTGCTTGATCGCAGCT<br>TTAAGACGCTGCTTTGTTGTGCTCGCAATTCCCATCGAAGTTGAACTCGTTGACGTTAGTGGTCCATTCAGCGCAGAAGTCGAC<br>GACGACTTCGCGGATTTTCCACCAAGTGCTGGAGTCGTTCCAGGAGTCGAGGAAGCCAGGTCCATAGTCTCGTTGGCACCCGT<br>AACAACGGTGACCGGAGTCGAGAGAGCAATGGGAAACGCGTCGCCGTTCGGAGACTTGATGACGACTTGGAGGAAGCAGATC<br>CCACCCGACTCCGGAAGCATCGTATTTGCCGACGTAAGAACCGAGTTGAGAACGGAAGTCGACGTCGTCAGACTAGATGTCGT<br>GGAAACCCCAAGAACCATCGAGTCTTGACTCGAGTCGTGGTCGAGCGGCGAGTCGAGGTCTTGAGATATGCCGTGTGTCGCCG<br>ACAGTGCATCGCGTGACTGCGACTTCAGTAGGCCCGAAGTCGTGTTGGACTTCCACCGCTGGAGCGGTTTTCCGATGGTAGTGT<br>GTATACCGGTTGGACTCGGCAACGGTATTCTTGATGGCTCACTTCTTGCAGCTGCTGAGCGCACCTCTTCGGTGACGGTAGAGC<br>CACCCTCGTGGTTCGGTCCGGGCAAAGGAATGTAGTTGTCAACGCCAACTCCCGATGTCACGATCCGCCTAACGGGAATGAGC<br>TTCTCTCGACGATGGTTCTCATCGTCCAGTAACAGCCCAGGATTTGGAGTGTTTAAGACGGCCTCGTTGTCACACATAGGCCCT<br>GGAAATGGACTCGACTCGTGCAGGTCATGGGCTTTTCCCTCGGCAAAAGCCTTCTTGAAGGCTTCCTCGAAAGGATTTTTCGAC<br>AGTTCATGGAAGAGACCGATCTCCTCACAGTTGCGTAGGAACTTAGTGGGAGTTGGAGTCTGATCTACAAAGGTGATGACAGT<br>GCCCGCCCTTGCATTGCCCGATGTCAGAGCAAGGCACATTTCATGCTTTGCCTTGTGCACTCCAAGGTGGTCCTCATTCGTGAA<br>GCGCATTCCACATCCTGGCTCTGTGCACTCGAATGGTCTGTCATCGCCCATGACAGCGATTCTCAAATTCGCAACGCAATTTGA<br>GAATCGTCCCGGATTACCGGGATGGCAAAGAATTAACGTCCAAAAGGCTGAAACAGTCTAAAACTCAAATAATAATCCAATA<br>TCCCAAAAGTGTTCGCAAAGGTTATTCTCACAAAATTAGACGCCATGTTGTCGTGAC
A<br>T<br>F<br>7	AAGCTGAATCTGCCAAAACAGTCGAATATGACAGAACACGAAAAATCATTTAATAACACCGATTCTGCAAACTTTGTAAAGGG<br>CGAGCAACACAGAAGTAGTAACCATAACATTTCACCTCTGCAGATGCCAATGATACCTACCTTTGTTGCGGACCTCACCCCAA<br>CTCCAACGCGTTTTCTCAGGAGTTGCGATGAGGTTGGGCTTTTCAATGAAATCTCCAAAGGCTCATACAATGAAGATTTCAAAC<br>GACAACACTCCACAGACGAATCGAATTCTGTTCTTTCGCAACTGCCTTCGCATTCTCAAGCTCCAATACCATCTGTCATTTTAG<br>AGTCAATTGCTGATGATAATGAAGTGGATGAAGAGGAGGAGGAAGAGGAGGAGGATGATTCGGCTGCTGCCCTGGAAGACCA<br>GAGTGCCAGTAATTTTGCAGTTTCTATGATGGAAGCCAGTTCACAGCAGCTGATCTACAGTGCACCACAGGAATCTGCAGCAA<br>ACACACCAACCGAAGTGGTGATACTGGAGTCTCCAGCTGAGAGACCACATATCCAGGTCCTGCTTCAGCTTCCCCAAGGTGGT<br>TTCGTCCCCATCAACATCCCTGCTGATGCTTTGGCCCATGCCACCTTCCCTGGTAGCGCTCTCATTGGCGTTGGGTCACCAGTGA<br>CACAAGTCGTATCACCGCTCGTAGTAGCACCCGAAATTGCCAGTACAACCTCATCAACATCAGGAATTACCTCAACCACAAAA<br>CAGAAGCTGAAAGCGGCCGTACAACAGAACATACAGCAGCAGCAACTTACTCACCACCAGCAGCTTCCGCAGCCAATCGCCA<br>TAGCACCAGCATCTGTGAGTAGGGTAGCTCTGCCAGACGTATCATCATCGTCATCCTCGCACGGCTATGGACCAAGCGTCTCA<br>AAAACTCCGAAATTGTCCGTAAACTCGACAGCTGCACCGTCTGACATCATGAGAAGTCTATCGCAGCCTTTGTCTTCACAAAGT<br>TCAGATGAAGAGGAAGGCGATGGGAAGCGAAAGAAGTTTCTCGAAAGTAACAGAGCTGCAGCCAACAGATGTCGGCAGAAG<br>AAGAAACAATGGATTAACAGTCTTGAGGAAAAGACGAAACAACTGCAGGACGTCAACAAAAGTCTCACAACTGAAACATCAT<br>CGTTACGAAGAGAGATTGCTCATCTGAAGCAGGTCCTCCTTCAGCACAAGGACTGTCAACCATTAACTATCATTTCAAATGTTC<br>GAACAGATAGCAATTTTTGACTTGAGAAGCCATGATTCACATCCTGACAGATCAGTAATGGCGCAATGGGATTACTAATTAAT<br>AATCATGAATCCAGCAAGGGATACTGATGAACATTCACCAATTATACAAATGAGAACTTTTGAATATTTGCAAGTCTTCTGGA<br>AGCTGCACATACAAGGAATGGTGATGGATATTCGTGGTTGACCAATGGATGTTGAATATTCATCGGTTATTCAAATGATGGAG<br>AATAAGTAAATATTCACTGACTCGAAGTTCTGGTCATATTATCGGTGACAGATTTTCTTAAATGTTTCTGCAACTTCTGGTAAA<br>GACTGTTGAGGATTATTTATTGACAGGAGATATCTATTGTATATGCATTGTGCACTGTGAATGTTATTGAGGTGTCGGTGACCA<br>TTTTTTAGACAATTTGGACCGTAATAGACGGACAATGGATAATATTAATTCCTTGTGAACACCGTGGTAATGGGACAGTGCTAA<br>AAATGTCATGCATTGAAGGTGTATTGAAGGTGTATGTGTAGTAGTATAATTATGAAAATTGTTAATGTTTTATCAGACCTGGTG<br>ATGAAATCATCTAATCATTCTAATAACTTCTATTATATATAGTGCTATTATCATAATGCATATGCCATGAATTCATGGTCAATCC<br>TGTAACGAAGGTAACACATTTTTGGCATTGAGCAATACAACTTTGTATTTAAAATTTTGTTTTTTGT
**C**<br>**R**<br>**E**<br>**B**	CTCTGTTTTGCCTTATGGGTAGCGGTCAACTTCTCCGTTTTATTTTCAATAATAAATTCTACTTGAGCTAATATTCTCTTATAAAT<br>CGTTCATGTAGTATGGCAGAGCAGTGCGACTTGTCAGTGGACGTCATTATAGAATATTCAGATTGGATGATACGCAATGCTGA<br>TTGTTGGCTGGTTGTGCAGTGACTAAAGTTCATTCTCAGGCTATCACAACCGAAGCTTCAGGTGTGCTGTAAGGACTGATTACA<br>CCATACATTCAGCAATAAACGACCAAGTTCGCACTTTTCTACATTTAGACATAAGCAAAGGTCTTTGCAAGGTGTTTATCGAGC<br>TGGCGTCGAATACCTCACTCAAGTACAGCATCCGGAGGAAGAAGATGGATGACAACTCAAGCGATGAACAACTGACGCAAGA<br>CAGTAACGACACAGGCGACGAAGCTATGCCTTCAATAGGACTTGGCGGGCAGCAGTTCCAGATCGTCTCGCAAGACGACAAC<br>GGGGAACCAGGCATTCACACGATCACGATGAGCAACGTCTCCTCGTCTGGTGGCTTGCCATCCTCCGGACAAACCATCCTACA<br>GTATGCTCAGGGACCTGATGGACAATATTTCATTCCAGTACATCAGTTAAGTCCGTCTGATGTTGTCTCGCTGTCCCAAGGTCT<br>CGTCGTTACGGCTGGCGGTGTTGGTCTCGACTCCGCAACGCAGATGGTTGAGGAGGCGTCGAGAAAACGAGAACTTCGTCTGC<br>TTAAAAATAAGGAAGCCGCAAAAGAGTGCCGACGGAAGAAGAAAGAATACGTCAAATGTTTGGAAAATCGCGTCGCGGTGCT<br>GGAAAATCAGAACAAGACCTTAATTGAGGAGTTAAAGTCGCTGAAAGAACTGTACTGCCAGAAGGACTCGCATAGCTAGCCT<br>GCACGCCGCCTCGTATGACAATGGACGACTTTTCTAAATGTGCTGTACAGACAATGGTTGGGTCGTTGAAGAAGTTTCTGTGAT<br>CGGCTGGAATAGAATAAAAAATTACTCTGCCTTATGACTGCTGGTGTGCGTTCGAAGTTTAGGATCACGATCAGTTCGCGCGTC<br>ACGAGCAGCTACTAGGCACGGCAAGGAACTGCTTCGAAATTATTCTACCGAGGACCAGTGTGGCGTTCCGATGTACATAGGTA<br>GATTTTTTCGCAGAATTTATTATTCGCATGTTAAGGTTTTTCTCATTCGTGTTTGGTTCTTTTATGCAGACTGATTTGATATGTTC<br>GAGAGATCTAGGCGCTTTCTTGAGCACTTCCAGAAGCAGCTTTTTTATTTGTTTGAATGGGTTTTGAGCTCACTCAATAGTTACA<br>TAAATTGATGTGTCATTTTGTTTACTTACCGTCAGAAACCTTATTTAACAGGAGTTGCGAACAGTGCGGCGTGAATGATTCTGT<br>GCTGTGTAGAGATGACAACCTACGAGTTTGATGCTTTATATGAAAATGCGTTTCAAAATGTGTAGAAATGCAGATATATGACT<br>AAAAGTAATTTGAGTGATTTCACGTATGGCTTACAAGTGTGAAAATGACACGAGTGATTAATATTGCTTATGCAAGAAGCGTC

	TGATCAGTTTGTCAGGTGCTGATAAAAGATCTTCAGTGGCGCATTCTTCCTTTTTTGTTGCACTTTCAACTTTCCTTACCTTTCCK<br>GTGAAATCAACTTCTTATGCTGACTTGATAATTTTCCTGCCTAACATCTTCCGTTTCTTGGTGAATATTTATGCACTTAGGAATT<br>TTGCTAAAGAGCAATCATAACGCTGTGTAATGTGATTGGAAGCCAACACAAAAGTGKGGAATTTATGCAYTCTTRTAGTGGAY<br>TGGGCATTGTAATAGGTGGTGAGTGCAAGTAAAGAGTYRACTAGGTTAMGCAAAGGCAAGGATTTATTCTATGTGGCGTTAAA<br>TAAGTGTGGCTGTCATGCAGTGGACTGGTTGGCATCAAATGAAAAGTGACRGTTTGAGTACAATTAATTTATTAAACATGATG<br>GAATGATTAGCTAGATGGAACGAGAAATGAGATGTGGTGCTGAAGAATGAAAACAACCAACATGTGATGAAGCCAAAGATTT<br>ATATAAAACAGAGATGGATTACACAGAAATTTTCATACATTGCAAATTGCTGTTTGCGTGTAGCCGAGTGTGTGTATTTTATCT<br>CTTTAATAGAGGGTGGGGTAAGWGTGTAAAGCGAACTTGATGCCATAATTGTTTTCTTTCTGTATTTACTTTCCTTTAGCTTTTT<br>CAAGGAAAATATCTTTCTGCTTTGCTTGAAAAATCAAGCACTGTCTGTACCATTGTTTAGTTCTGGGCTTGTTTTACAACATCGT<br>AAAAACTAAAGCTTGACTTTTCATTTATTTATCACCAGTTAGAGCAGTGGGGAGTTGTGCTTCAACTTCACTGTTACCTCATTTG<br>TCTTATTTTTCGTAATCTTCATTGTATTCTGTTTGAWCAGTAACAGATTCACTGAGAGAATAATAYGCGAAAGCATGGCGTAYC<br>CGCAAAATTGGCAGGMATGTTTTCCCTTATTGTTTTATTGCATGTGTCAACGTATTGTGTCCTACTTTATATGTTGCCGAAGATT<br>AAATATAGTACAACCCAGCTATGTGATATATGGAGCCTATTTAAGGTATTTGTTCGTATGACATGTATATATTTATAGGGTAGT<br>ATGCTCGGGCATTGGACTTYRTCCACTGCATTTGATTTTGYGAAAACAAGTACTTGGCTTTGGTTCTGAAAAATTTGAAAAAGA<br>AGTAATTTTTAAAATTGTTCTTGGAAGTRAAAATGTACKGAAAACATTCTTAACAATTTATCAGATGAAATTRGCTATAAATTA<br>TTTAATTGTAACACCTGTCCTGGTTTGTTGKGTGTTSACCAGAATGTGTGCGTAATATATGTATGTGTGTGT

1.4 Table S4. PCA contributions to Dim1 and Dim2

Table S4. Contributions to PCA from dimension 1 and dimension 2.

	Dim1		Dim2
**ATF7**	13.893138	**P_AMPK**	22.795757
**CREB**	13.891219	**HSP70**	19.368725
**ATF2**	12.781943	**MT1**	15.300463
**TLR1**	12.007888	**MT2**	11.126926
**Zinc**	10.210923	**Protein**	9.928185
**Protein**	10.052578	**TLR2**	7.841839
**Calcium**	8.369727	**TLR1**	5.309922
**Carbohydrates**	6.28673	**Zinc**	4.299573
**MT1**	4.562047	**CREB**	2.548627
**TLR2**	2.606753	**Carbohydrates**	1.086745
**HSP70**	2.124547	**ATF2**	0.224722

**P_AMPK**	1.715029	**ATF7**	0.164610
**MT2**	1.497478	**Calcium**	0.003907

2 Supplementary Figures

2.1 Supplementary Figure 1

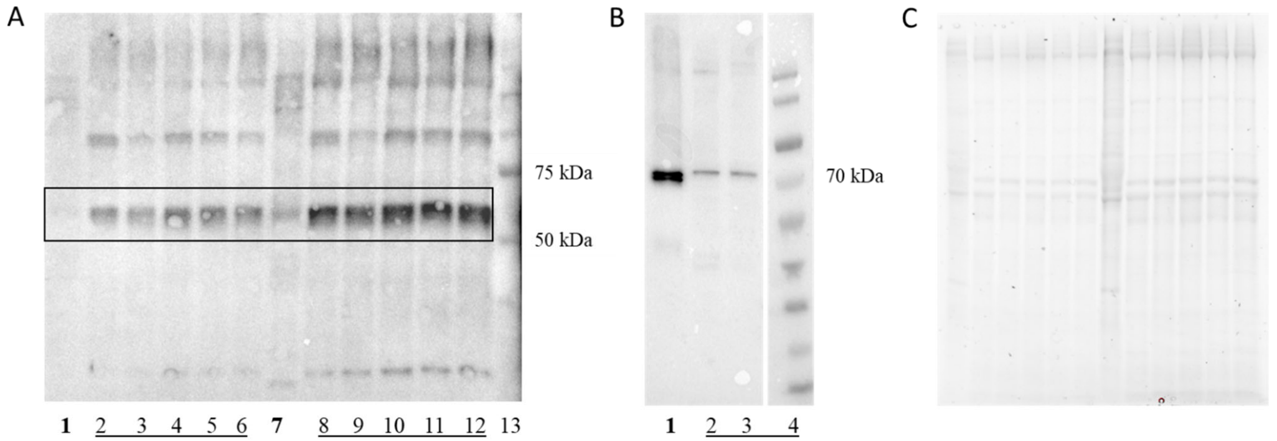

Fig.S1. Positive controls for Western Blots and loading control

(A) P-AMPK (63 kDa). Mouse protein (from NIH 3T3 cells) was used as positive control. To test the specificity of earthworm protein from tissue sections against the antibody for P-AMPK, mouse and earthworm protein was loaded onto the same gel in a test run. Lanes: 1 NIH 3T3 30 µg, 2 – 6 *L. terrestris* protein 30 µg, 7 NIH 3T3 60 µg, 8 -12 *L. terrestris* protein 60 µg, 13 Precision Plus Protein™ All Blue Prestained Protein Standards (Biorad). **(B)** HSP70 (70 kDa). Mouse protein (from NIH 3T3 cells) was used as a positive control. To test the specificity of earthworm protein from tissue sections against the antibody for P-AMPK, mouse and earthworm protein (40 µg) was loaded onto the same gel in a test run. Lanes: 1 NIH 3T3 2-3 *L. terrestris* protein 4 PageRuler™ Prestained Protein Ladder, 10 to 180 kDa (Thermo Fisher Scientific). **(C)** Stain-free gel that has been activated using UV light. Each lane was selected as a whole to detect whole protein content, which was accordingly used as loading control and for normalization.

2.2 Supplementary Figure S2

P-AMPK

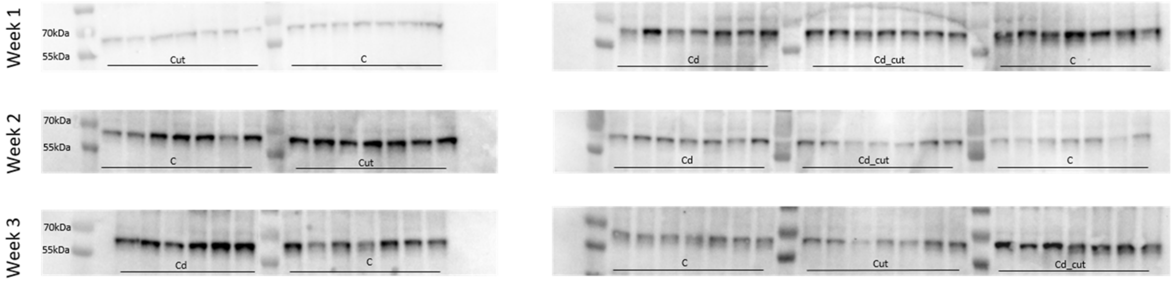

HSP70

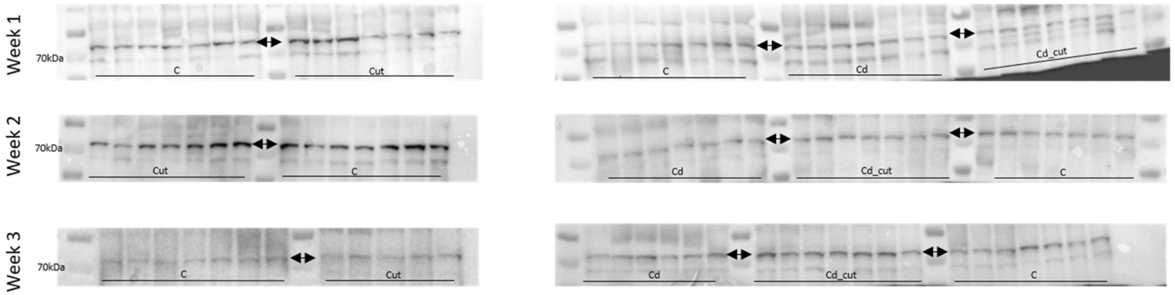

Fig.S2. Western blot membranes of all groups used for analysis

Each treatment group was loaded on a gel together with the respective control. Two western blot gels were necessary to load all samples from one week. For analyzes, each band was normalized to the loading control (total protein) and the mean value of the respective control.

2.3 Supplementary Figure 3

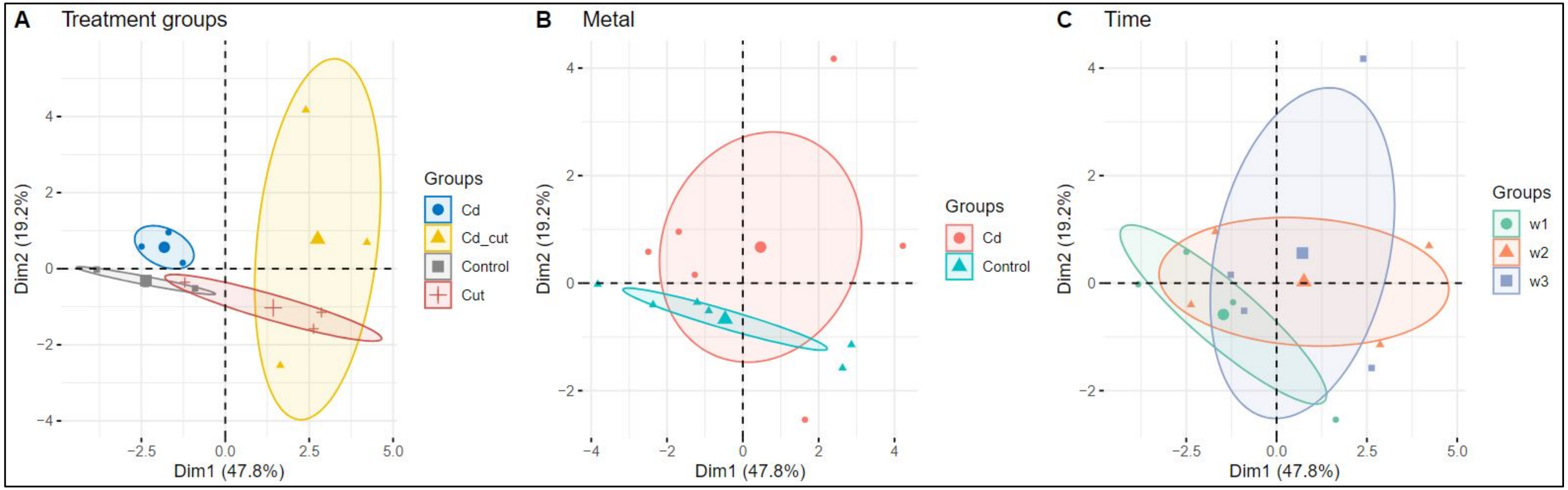

Fig.S3. PCA means of all four groups

Principal component analysis (PCA) based on the mean values: **(A)** Treatment groups – unharmed exposed to 50 mg/kg $CdCl_2$ (Cd), amputated exposed to 50 mg/kg $CdCl_2$ (Cd_Cut), Control, and amputated individuals in non-contaminated soil (Cut); **(B)** Metal – Cd, and Control; **(C)** Time – individuals at week one (w1), week two (w2) and week three (w3). The percentage of variation between the treatment groups for dimension 1 (Dim1) and dimension 2 (Dim2) are shown. Each ellipse presents a confidence level of 0.95.

2.4 Supplementary Figure 4

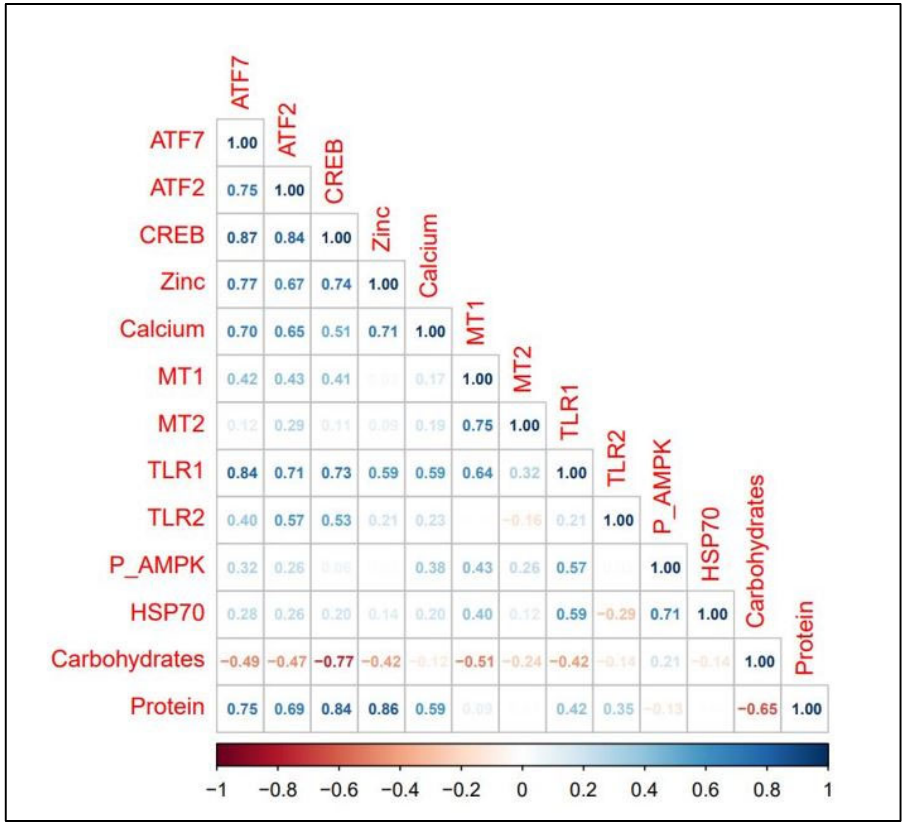

Fig.S4. Correlation analysis

Correlation analysis of all factors based on the mean values.

2.5 Supplementary Figure 5

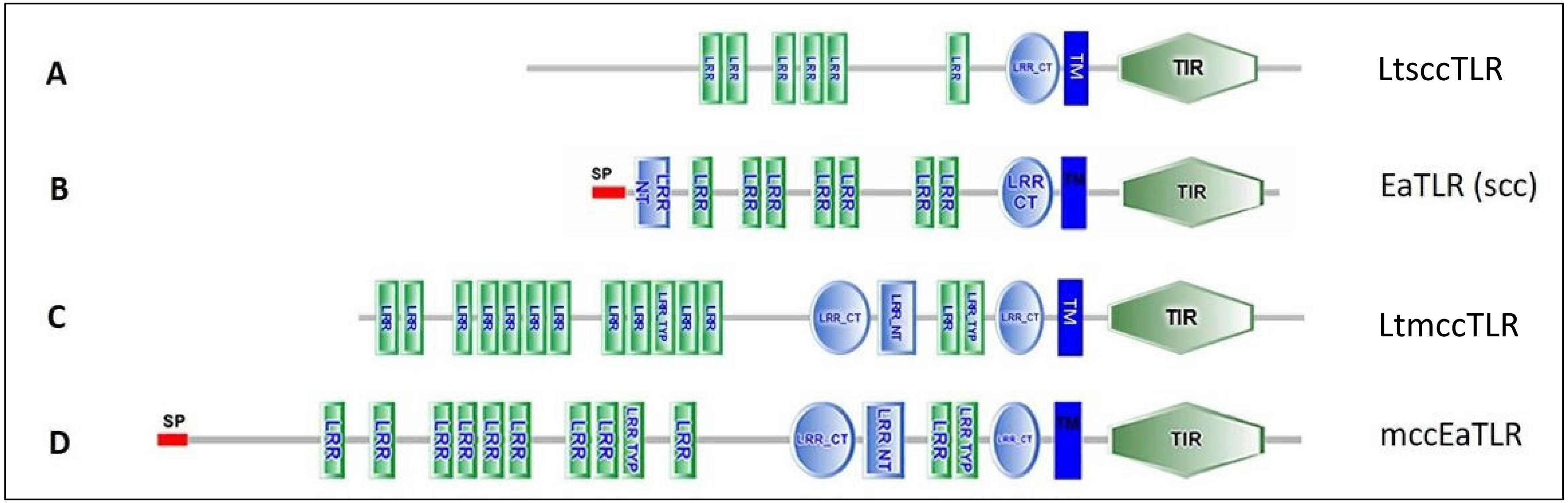

Fig.S5. TLR domain structures

Protein domain structure of two types of *L. terrestris* toll-like receptors (TLRs). (**A**) Protein domain structure of LtsccTLR, (**B**) typical sccTLR (*Eisenia andrei*; NCBI: JX898685), (**C**) LtmccTLR, (**D**) typical mccTLR (*Eisenia andrei*; NCBI: CZQ50134). SP, signal peptide; LRR, leucine-rich repeats; LRR Typ, typical LRR subfamily; LRR-NT, N-terminal LRR domain; LRR CT, C-terminal LRR domain; TIR, toll interleukin-1 receptor domain; TM, transmembrane region. Different domains were identified using the SMART^{TM} online tool.

2.6 Supplementary Figure 6

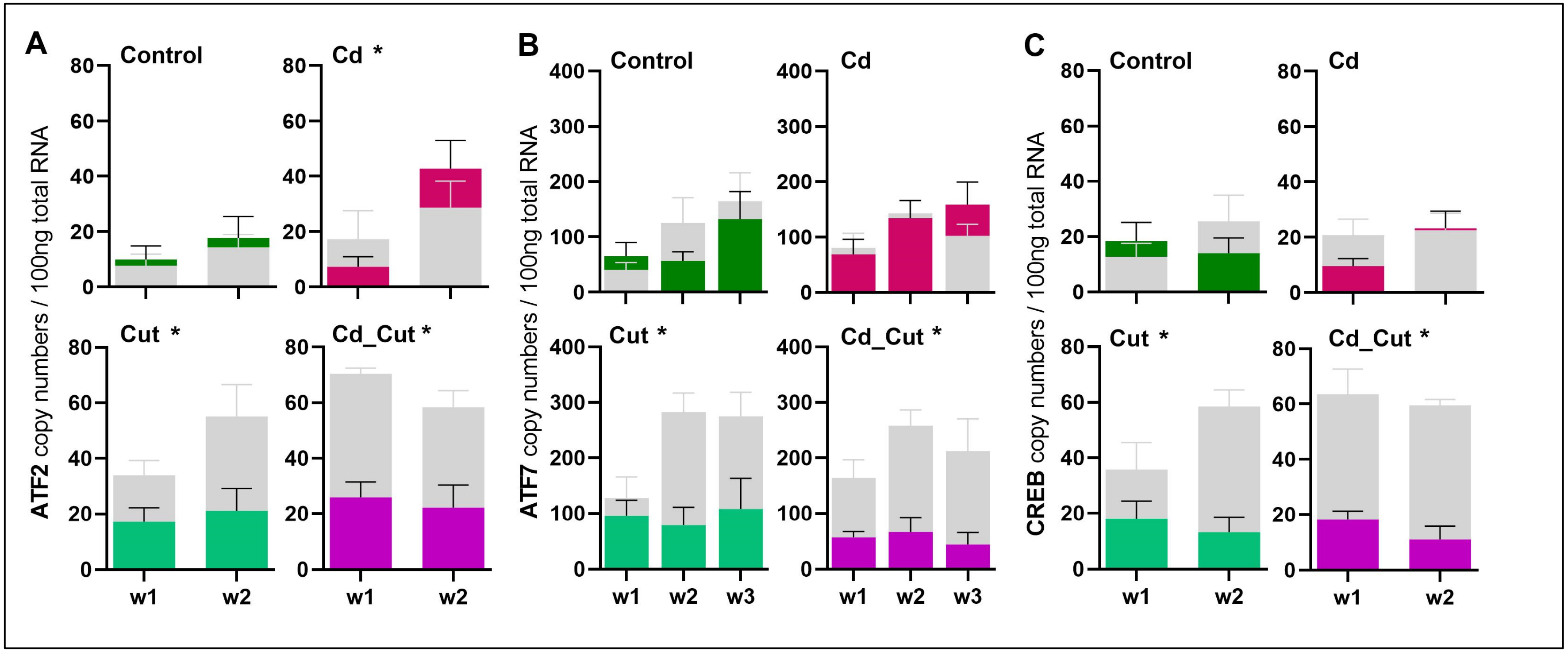

Fig.S6. Tissue 2 ATF2, ATF7 and CREB

Comparison of absolute mRNA copy numbers in Tissue 1 and Tissue 2 of (**A**) activating transcription factor 2 (ATF2), (**B**) activating transcription factor 7 (ATF7) and (**C**) cAMP response element-binding protein (CREB) in *L. terrestris* tissue from Control; exposed (50 mg $CdCl_2$/kg dry soil (Cd)); amputated (Cut) and exposed amputated (50 mg/kg $CdCl_2$ (Cd_Cut)) individuals at week one (w1), week two (w2), and week three (w3). Grey bars represent gene expression in Tissue 1 (including regenerative tissue) and colored bars represent gene expression in Tissue 2 (anterior section, no regenerative tissue). Stars indicate significant differences between Tissue 1 and Tissue 2 using mixed-effects analysis ($p < 0.05$ (*)). Mean values ± SEM are presented.
